# Predicting economics student retention in higher education: The effects of students’ economic competencies at the end of upper secondary school on their intention to leave their studies in economics

**DOI:** 10.1371/journal.pone.0228505

**Published:** 2020-02-05

**Authors:** Michael Jüttler

**Affiliations:** Department of Economics, University of Konstanz, Konstanz, Germany; Universidad Nacional de Educacion a Distancia (UNED), SPAIN

## Abstract

Despite the importance of acquiring economic competencies at the secondary level and the worldwide popularity of economics in higher education, there is almost no research on the effects of economic competencies on economics student retention. Based on a longitudinal sample of 538 high school students in Switzerland, this study provides the first results on this topic. The longitudinal study took place from 2011 to 2016 and comprised two points of measurement. Economic competencies were measured multidimensionally and comprised knowledge and skills, as well as attitude, value-oriented dispositions, interest and motivation. Different student retention models were adapted and combined to explain student retention in the field of economics. According to these models, students’ academic and social integration are key mediators to predict their retention. Based on these theoretical explanations, structural equation modelling was then used to test the long-term effects of high school students’ economic competencies at the end of upper-secondary school on their retention in studying in the field of economics. The results show that economic competencies predict economics students’ academic integration (as measured by grade point average) but not their social integration. Additionally, the data confirm that academic and social integration are strong mediators for their retention. In addition to economic competencies, students’ cognitive abilities, prior schooling (school grades, school profile, and school type) and perceived family support predict student retention in economics. Overall, economic competencies account for a substantial proportion of the variance in student retention. Against this background, the results indicate that fostering high school students’ economic competencies plays a crucial role in their study success in the field of economics.

## Introduction

The description and explanation of study success [1] has received increasing attention within the psychological, social and educational research for many years [2]. Although high school graduates strive to choose a field of study that fits their interests and skills as best as possible, many students still show insufficient performance in, are unsatisfied with and drop out of their university study program [3,4]. With respect to this, federal statistical data show a relatively high average dropout rate of approximately 30% (e.g., in countries of the Organisation for Economic Co-operation and Development (OECD)) [5,6]. Successfully graduating from university or college leads not only to higher personal long-term financial well-being but also to a higher return on investment for society [7–9]. In contrast, dropping out often leads to high monetary and non-monetary costs for individuals, institutions and society [10–13]. Against this background, student retention in higher education continues to be a major concern in various countries worldwide [3]. There is a long and broad research tradition that attempts to identify the individual, institutional and social variables that predict student retention and suggests that among other factors, general academic skills, intrinsic motivation, academic self-efficacy and social support (e.g., from family) are strong predictors [2,7,14]. However, on average, the aforementioned predictors explain only approximately 20 to 25 percent of the variance in student retention; a significant part is still unexplained. One reason is that most studies do not consider domain-specific characteristics, e.g., individual domain-specific skills. Therefore, researchers suggest considering specific characteristics of different fields of study [15].

Economics has become the most popular field of study in higher education worldwide, with over 20 percent of students studying this subject and graduating with an economics degree [16,17]. At this point, it is important to note that in this paper, related courses of study, such as business administration, accounting, etc., are subsumed under "Economics". Already twenty-five years ago, researchers have emphasized that an economics education in schools is an indispensable part of individuals’ skills and abilities to address typical economic issues, to shape their own lives, and to foster their social participation within a modern society [18]. In addition, they noted the considerable lack of economic competencies within many (western) countries. Since then, many researchers have suggested the positive effects of an economics education at a secondary level on students’ economic competencies [19–21]. However, no longitudinal study has examined the effects of students’ economic competencies at the end of upper secondary school on student retention in economics. Against this background, it must be questioned whether high schools appropriately prepare students to study in economics. The present study addresses this question by using data from a representative and longitudinal sample of Swiss students. In Switzerland, there are no systematic constraints that prevent students from switching to another field of study or dropping out, e.g., high monetary costs or legal restrictions. Therefore, it can be assumed that dropout decisions and intentions represent unadulterated processes that are largely shaped by students’ individual characteristics (e.g., skills and interests). Additionally, upper secondary students in Switzerland gain at least a basic education in the field of economics. Therefore, they are exposed to this domain through secondary education before deciding to study economics. Finally, transition and dropout rates in the field of economics at universities in Switzerland are internationally comparable [17,22]; thus, the results can be better applied to other countries.

The theoretical background consists of three parts. First, student retention in higher education is explained based on two theoretical approaches. According to these approaches, a brief literature review of the key findings in this context is provided. In the second part, economic competencies are defined, and the reasons for their importance to higher education are given. Finally, the transition from school to university in Switzerland is briefly described to give an overview of country-specific characteristics and to address the question of the replicability of this study in other countries. The overarching research question and hypotheses are derived from these considerations. After describing the study design, the used instruments and the overall analysis strategy to answer the research question, the results from the empirical analyses are presented. The paper is closed by discussing the results and drawing conclusions to be considered in future research and practice.

## Theoretical background

### Student retention in higher education

Student retention has received comparatively high attention from a theoretical perspective [23]. Among the first and still most popular models are Tinto’s *Student Integration Model* [24] and Beans’ *Student Attrition Model* [25], which have been repeatedly revised [26,27]. Both models describe the processes that lead to dropout intentions and decisions in higher education. Additionally, both models describe student dropout in general and do not focus on a certain domain. Tinto argues that students enter university or college with different intentions, goals, commitments and expectations (“initial commitment”). These differences can mainly be traced back to students’ individual characteristics (e.g., gender, study skills), prior schooling (e.g., school grades), and family background (e.g., socioeconomic status (SES)). At the university level, Tinto differentiates between the academic and social systems that students are a part of and should be integrated into to prevent attrition. Academic integration is mainly described by students’ academic performance (e.g., university grade point average (UGPA)), whereas social integration is described by interactions with college or university society (e.g., peer group interactions). The probability of being integrated into these systems strongly depends on students’ initial commitment. Finally, academic and social integration are the core mediators that explain students’ subsequent commitment, which ultimately determines their decision to drop out. Bean revised Tinto’s model [26] by adopting a more behavioral than cognitive approach. Accordingly, he places a stronger emphasis on external factors [28]. First, Bean [26] includes considerations of the theory of planned behavior [29]. In this regard, there is empirical evidence that the effects are canalized through the intention to drop out, which functions as a key mediator for the realized dropout [30]. The two theories overlap in many facets and adequately explain student retention (ibid.). Researchers strongly recommend considering both theories [15,28].

### Explaining study success: A literature review

There is a long research tradition regarding the prediction of students’ study success [2]. Many meta-analyses and reviews have been conducted on this topic and offer a comprehensive overview. Table 1 summarizes the main results of these meta-analyses for UGPA and student retention. The most prominent predictor is the overall high school grade, which is the best predictor of the overall university grade [31]. A similar predictive validity can be found for college admission tests, such as the Scholastic Aptitude Test (SAT), American College Testing (ACT) or the Graduate Record Examination (GRE) (ibid.). Additionally, many researchers find intelligence to be the most important predictor of academic performance [7]. Finally, SES was found to have rather small correlations with UGPA and student retention [32].

**Table 1:** Results of meta-analyses regarding the explanation of UGPA and student retention in higher education.

Predictor	ρ Retention	ρ UGPA	Source
***Traditional correlates***			
Average school grades	.25	>.40	[15,31]
General study ability tests (e.g., SAT or ACT)	.14	>.30	[15,31,33]
Subject-specific study ability tests (e.g., GRE subject tests)	.39	.48	[34,35]
Intelligence	.21	.21-.39	[31,36,37]
Socioeconomic status (SES)	.23	.16	[15,31,32]
***Psychosocial contextual correlates***			
Goal commitment/academic goal	.34	.12-.18	[15,31,33]
Institutional integration/commitment	.26	.03-.12	[15,31]
Social support	.26	.09-.11	[15,31]
Social integration	.22	.03-.14	[15,31]
***Further (study) skill factors***			
(Subject-)specific interests	--	.28	[38]
Achievement motivation	.34	.28-.30	[15])
Academic self-efficacy	.36	.28-.50	[15,31,33]
General self-concept	.05	.05	[15])
Academic-related skills	.37	.16	[15])
***Big Five Personality Traits***			
Neuroticism	-.06	.02	[36,39]
Extraversion	-.02	-.01	[36,39]
Openness to experience	--	.12	[36]
Agreeableness	--	.07	[36]
Conscientiousness	.03	.22	[36,39]

ρ: true construct correlation corrected for measurement error; UGPA: university grade point average

In addition to these traditional correlates, there is broad research on psychosocial and study skill factors (PSFs) [15]. Based on this meta-analysis, goal and institutional commitment, social support and social integration play a crucial role in student retention. Furthermore, academic skills, achievement motivation and academic self-efficacy show strong correlations with UGPA and moderate correlations with student retention. These results are also supported by other meta-analyses [14,31].

Furthermore, many researchers suggest positive effects of psychological variables such as interest, intrinsic motivation, attitude, and personality traits on facets of both academic and social integration [2,36,38,40,41]. For social support, relationships with parents and friends as a common predictor of the academic achievement, critical thinking and social-emotional well-being of students in higher education has been identified [2]. A comprehensive overview of further predictors is also provided [7].

Therefore, there is strong empirical evidence of the importance of students’ general academic skills (including academic self-efficacy), overall school grade, motivational disposition, social support and goal and institutional commitment to predict student retention. Against this background, the models by Tinto [27] and Bean [26] are well supported. However, the research on moderating effects is lacking. Domain-specific characteristics are, with some exceptions [34], mostly neglected. Additionally, theoretically well-grounded empirical research is rudimentary. No longitudinal studies have considered the role of economic competencies at the end of upper secondary education. It is thus unclear how students’ economic competencies predict their study success in economics in higher education.

### Definition of economic competencies

A widespread understanding of economic competence is that it represents people’s skills and abilities to act as informed citizens within modern societies by being able to understand typical economic issues [18,20]. This understanding is also known as economic literacy. The definition in this study follows this conceptualization. Based on the general definition of domain-specific competencies [42], economic competencies comprise

1. **economic knowledge and skills** required to be able to solve economic issues and to judge suggested solutions to economic problems and
2. **the motivation** to address and **interest** in economic issues.
  The definition can be further elaborated [43,44] to include
3. **an attitude towards economics** and **a value-oriented disposition** that involve reflectively solving economic issues and appropriately judging certain solutions.

Economic knowledge and skills form the core, as they represent an important prerequisite to the ability to solve economic issues. This definition is similar to many other definitions [45,46]. However, apart from knowledge and skills, most studies do not implement further facets of economic competence [47, 48]. In the present definition, interest, motivation, attitude and a value-oriented disposition are considered and theoretically separated from one another. While motivation represents students’ intrinsic motivation to solve economic issues [49], interest in economics is more object-oriented and describes students’ dispositional preference for solving these issues [50]. An attitude towards economics describes students’ willingness and ability to activate an economic perspective [43]. Finally, a value-oriented disposition describes students’ ability to solve economic issues in a morally, socially and ethically appropriate fashion. If this orientation is not considered, it is cautioned that economic criminals would also be counted as economically competent persons [43].

### Relevance of economic competencies for higher education

Considering the growing complexity of the economic context in modern societies and the increasingly connected global system, the development of students’ economic competencies has gradually become an important part of general education [51]. Walstad [20] pointed out “that the best opportunity for economic education occurs before graduation from high school”. Additionally, there are strong arguments for the relevance of economic competencies (or economic education) for higher education. First, there is empirical evidence that a high school economic education helps students when studying economics in higher education [19,21,52,53]. Therefore, the suggestions by Walstad [20] are also crucial for higher education. Second, approximately one in four students within higher education is enrolled in economics [16,17]. Against this background, fostering economic competencies at the upper secondary level becomes relevant for preparing a large number of students. Third, a main aim of upper secondary schools that prepare students to study at a university is for students to have proficient study abilities. Considering that approximately one in three economics students drops out before graduating [17], fostering economic competencies is important to better reach this aim. Finally, considering that general academic skills and school grades explain only 20 to 25 percent of the variance in student retention [15], domain-specific characteristics should be focused on in more detail. Many studies provide empirical evidence on the positive effects of a prior economic education on the economic knowledge of first-year economics students [21,54,55]. In addition to the effects of economic education, higher education research has long observed the positive effects of students’ math skills, admission test scores, GPA in preparatory education, and motivational orientation on study success in economics [56]. Finally, there is a broad discussion on the gender-specific effects of economic competencies on study success in economics (ibid.). In this regard, researchers have found that female students in economics are more motivated but less confident about their study performance (ibid.). However, these differences do not affect study success in economics. The results of recent meta-analyses reveal that the gender gap in economics has decreased annually during the past few decades and is often overestimated [57,58]. However, most of the aforementioned studies use cross-sectional data of undergraduate students at universities, do not refer to achievement tests, or do not consider indicators of study success (e.g., student retention or UGPA). Thus, it is not clear whether the higher economic knowledge of first-year economics students results in higher study success. Additionally, none of these studies refer to students’ economic competencies at the end of upper secondary school to measure the effects of these competencies on study success in economics.

**Fig 1:**
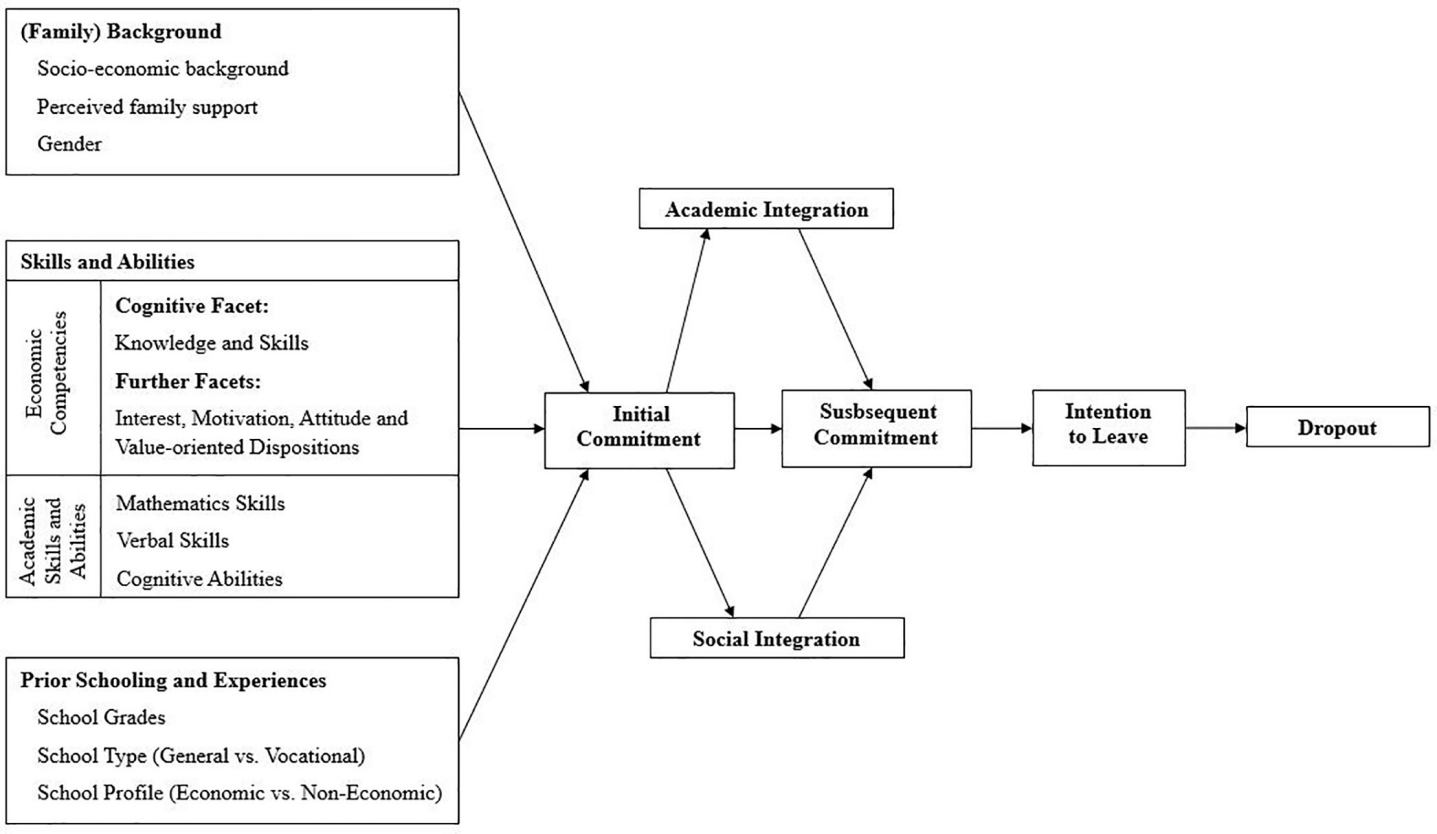
Theoretical model to explain students’ dropout behavior following [24–27].

To analyze these factors, the present study integrates economic competencies at the end of upper secondary school into Tinto’s *Student Integration Model* as a predictor of students’ dropout behavior in economics. Furthermore, based on Bean [26], the focus is placed on the explanation of the intention to leave, which represents the most proximal predictor of dropout decisions. The theoretical model is represented in Fig 1.

### Transitions into higher education in Switzerland

In Switzerland, there are two major paths to university entrance: (1) Baccalaureate School (BS; academic track) and (2) Federal Vocational Baccalaureate School (FVBS; vocational track). BS graduates gain a general qualification for university entrance, and there are no systematic constraints regarding their major choices at the university level (except for medicine). In contrast, FVBS graduates receive only a domain-related qualification for entrance into universities of applied sciences (UAS) parallel to or consecutive with an apprenticeship, which enables them to choose a field of study that corresponds to their vocational profession (currently, these professions are engineering, architecture, and life sciences; nature, agriculture and food; business and services; creativity and art; and health and social care). Accordingly, most FVB students of economics belong to business and services.

Because FVB students have the possibility to stay in the vocational track instead of studying, transition rates into higher education strongly differ between BS and FVBS students: over 90 percent of all BS students are enrolled in a university or UAS two years after graduating. In contrast, approximately 60 percent of all FVBS students are enrolled in a UAS. All BS and FVBS students gain at least a basic education in economics during school, because the subject “Economics and Law” is either a basic or an advanced course. All BS students choose one advanced course in school. In addition to Economics and Law, other advanced courses are Ancient Languages, Modern Languages, Physics & Mathematics, Biology & Chemistry, Philosophy/Pedagogy/Psychology, Art and Music.

## Research question and hypotheses

Based on the aforementioned considerations, the following research question will be answered:

How do students’ economic competencies at the end of upper secondary school affect their intention to drop out when studying economics in higher education?

Although the theoretical model and most research regarding student retention do not consider domain-specific characteristics, it is expected that students with higher initial knowledge and skills in economics and higher scores on the further facets (interest, intrinsic motivation, attitude and value-oriented disposition) in relation to economics show a lower intention to leave. These assumptions are supported by findings on the positive effects of previous economic education [21,54,55] and further explanations of first-year students’ study success in economics [56]. In the latter study, typical predictors, such as mathematics and verbal skills, cognitive abilities, school grades, gender and SES, are controlled for to prevent the existence of statistical artefacts. However, since economic competencies in this study mainly represent cognitive disposition, it is assumed that their expected positive effect on student retention is mainly mediated by students’ academic integration and not students’ social integration. It is expected that other factors, such as family support and prior schooling (school type and advanced course), are predictive of social integration. Against this background, the following two hypotheses are tested:

(H1) There is a negative effect of economics students’ economic knowledge and skills at the end of upper secondary school on their intention to leave, which is mediated by their academic integration.
(H2) There is a negative effect of economics students’ motivational disposition related to economics at the end of upper secondary school on their intention to leave, which is mediated by their academic integration.

## Method

### Study design

The study was based on a longitudinal design with two points of measurement (T1 and T2). T1 was conducted in summer 2011 at the end of upper secondary school. Students’ economic competencies, cognitive abilities, and mathematics and verbal skills were tested in school classes (approximately 180 minutes). Furthermore, students were asked about further facets of economic competence (interest, intrinsic motivation, attitude and value-oriented disposition), their study aspirations and their demographic aspects (e.g., gender). T2 was conducted approximately five years later, in spring/summer 2016. Students were asked about their major choices, indicators of study success (e.g., UGPA and the intention to leave) and further variables (e.g., study program).

### Sample

The target population comprised all BS and FVBS students of the German-speaking part of Switzerland who graduated in summer 2011; this population included 10,091 BS students (584 school classes) and 7,150 FVBS students (417 school classes). From this population, 100 BS and 100 FVBS school classes (1,838 and 1,802 students) were drawn equally from the following four explicit strata (and randomly within each stratum): 1) BS students with the advanced course “Economics and Law” (BS Economics and Law); 2) BS students with another advanced course (BS Other); 3) FVBS students who belonged to a commercial profession (FVBS Commercial); and 4) FVBS students who belonged to another profession (FVBS Other). In addition, implicit strata according to gender, canton and class size were built. Of the 3,640 students drawn, 2,328 (150 school classes) participated at T1. There were no structural differences between the school classes that participated and the school classes that did not participate [59]. Finally, a group-specific weighting was necessary because the distribution regarding the explicit strata within the random sample was not proportional to the distribution in the population (a disproportionally nested sample).

Of the 2,328 students assessed at T1, approximately 1,600 provided a postal and/or e-mail address for a follow-up study. Overall, 1,300 of these addresses were still valid in 2016. Ultimately, 520 students participated at T2 (see Table 2). A comparison between the samples at T1 and T2 showed that BS students were strongly overrepresented. Furthermore, there was a positive selection bias regarding economic knowledge and skills (d = 0.47), mathematics skills (d = 0.47), verbal skills (d = 0.38), cognitive abilities (d = 0.36), further facets of economic competence (d = 0.19 to 0.27), and school grades (d = 0.17 to 0.22). Because of this attrition (unit non-response), individual weighting was necessary.

**Table 2:** Longitudinal sample (unweighted).

	Classes	Students	Gender		Age	
	*N*	*n*	*Female*	*Male*	*M*	*SD*
BS (Economics & Law)	36	188 (36%)	84 (45%)	104 (55%)	23.5	0.7
BS (Other)	41	179 (35%)	112 (63%)	67 (37%)	23.4	0.8
FVBS (Commercial)	34	69 (13%)	44 (64%)	25 (36%)	24.3	2.2
FVBS (Other)	28	84 (16%)	28 (33%)	56 (67%)	25.4	1.8
*Total*	*139*	*520*	*268 (52%)*	*252 (48%)*	*23*.*9*	*1*.*5*

BS = Baccalaureate School, FVBS = Federal Vocational Baccalaureate School, M = Mean, SD = Standard Deviation

Inverse-probability weighting is one common way to address dropout effects and was used to prevent systematic errors in the results due to the probability of dropout based on the observed variables [60]. Logistic regressions were used to calculate the individual probability of dropping out (coded as 0) or remaining enrolled (coded as 1) based on the dropout variables mentioned above. The reciprocal value of this probability was used as the individual weight, and ten values had to be trimmed to prevent overweighting [61]. The weighted sample is described in Table 3.

**Table 3:** Longitudinal sample (weighted).

	Classes	Students	Gender		Age	
	*N*	*n*	Female	Male	M	SD
BS (Economics & Law)	36	193	79 (41%)	114 (59%)	23.6	0.7
BS (Other)	41	1,204	748 (62%)	456 (38%)	23.5	0.9
FVBS (Commercial)	34	381	216 (57%)	165 (43%)	24.6	2.6
FVBS (Other)	28	534	165 (31%)	369 (69%)	24.6	1.8
*Total*	*139*	*2*,*312*	*1*,*208 (52%)*	*1*,*104 (48%)*	*24*.*2*	*1*.*8*

BS = Baccalaureate School, FVBS = Federal Vocational Baccalaureate School, M = Mean, SD = Standard Deviation

The only significant difference that remained after weighting was for economic knowledge and skills (d = 0.26). The analyzed sample comprised all students at T2 whose latest field of study was economics and is described in Table 4.

**Table 4:** Analyzed sample (weighted).

	Classes	Students	Gender		Age	
	N	n	Female	Male	M	SD
BS (Economics & Law)	34	85	23 (27%)	62 (73%)	23.5	0.8
BS (Other)	21	217	78 (36%)	139 (64%)	23.5	1.0
FVBS (Commercial)	20	187	77 (41%)	110 (59%)	24.8	2.5
FVBS (Other)	7	49	23 (47%)	26 (53%)	26.6	1.9
*Total*	*82*	*538*	*201 (37%)*	*337 (63%)*	*24*.*4*	*2*.*1*

BS = Baccalaureate School, FVBS = Federal Vocational Baccalaureate School, M = Mean, SD = Standard Deviation

### Instrument

To measure economic knowledge and skills, mathematics and verbal skills and cognitive abilities at T1, achievement tests in a multi-matrix booklet design were used. The further facets that related to economics and other variables (e.g., school grades) were assessed with a paper-based questionnaire. At T2, the students were asked about their educational decisions by using computer-assisted telephone interviewing (CATI). An online questionnaire was used to assess study success and further variables. CATIs were conducted by trained interviewers at a professional survey lab of the Chair of Empirical Social Research (with a focus on survey research) at the University of Konstanz (see https://www.soziologie.uni-konstanz.de/en/hinz/surveylab/). Table 5 provides an overview of the instruments used.

**Table 5:** Overview of instruments at T1 and T2.

Variable	Items	Reliability	Source
***T1***			
Economic Knowledge and Skills	111	0.75^1^	Internal Development [59]
Further Facets of Economic Competence^a^	29 / 3 parcels [62]	0.79^3^	[63,64]
Mathematics Skills	59	0.81^1^	[65]
Verbal Skills	91	0.81^1^	[65]
Cognitive Abilities	45	0.78^1^	[66]
***T2***			
Socioeconomic Status	2	0.79^2^	[67]
Support from Family	3	0.72^3^	[68]
Social Integration	3	0.83^3^	[69]
Intention to Leave	3	0.83^3^	[70,71]

^1^Item Response Theory (IRT)

^2^Cohen’s Kappa

^3^McDonald’s Omega.

^a^The four further facets (interest, intrinsic motivation, attitude and value-oriented disposition) were modelled in one dimension by using parceling [62], and interest and intrinsic motivation were modelled as one parcel. Altogether, 29 items were used for the one-dimensional model.

All variables in this table are based on a ratio scale.

Economic knowledge and skills: Although several tests on economic knowledge and skills were available, a new test was internally developed because this study (1) examined a different target group and (2) had a different understanding of the construct, and (3) previous tests had psychometric deficits [72]. The items were developed based on a comprehensive media analysis of approximately 1,400 newspaper articles with approximately 30,000 economic terms and concepts that could be allocated to business administration, accounting and economics. Finally, 111 items were used and could be subdivided into these three categories [59]. However, there were no empirical indications of the superiority of the three-dimensional model to a general-factor model (ibid.). To simplify the empirical model, a general-factor model was used. The items of this test are mainly presented as multiple-choice questions (five items are open-ended questions). The students received modified newspaper articles, which enabled the establishment of a proper context. Based on each modified newspaper article, the students answered four to eight items that partly related to this newspaper article. Altogether, 21 different newspaper articles were implemented within six test booklets (multi-matrix booklet design).

Further facets of economic competence: The items that measured interest (3 items), intrinsic motivation (4 items) and value-oriented disposition (9 items) were adapted [63] and transferred to the subject “Economics and Law” [64]; example items (translated) include the following: *Interest*: *“Within lessons in economics and law*, *I am often confronted with interesting issues*.*”; Intrinsic motivation*: *“Within lessons in economics and law*, *time often flies by*.*”;* and *Value-oriented disposition*: *“I think that solutions to economic issues also depend on the personal attitudes of those involved”*. To measure these items, a four-point Likert scale was used (1 = “totally disagree” to 4 = “totally agree”). To measure attitude towards economics (14 items), a translated and adapted version of the “Attitude towards Economics” (ATE) questionnaire [44] was used [73]. The items of this questionnaire were based on a five-point Likert scale (1 = “totally disagree” to 5 = “totally agree”; an example item (translated) is: *“I like to read articles about economic issues”)*.

Chosen field of study: The present study focuses on students’ latest field of study. Although the effects of students’ competencies at the end of upper secondary school on study success can be expected to be stronger when considering the first chosen field of study, indicators regarding study success were expected to be most valid when asking students about their latest field of study. This is because it can be assumed that students can remember this study period more easily than periods farther in the past. To categorize the field of economics, the official categorization systems of the Swiss Federal Statistical Office [74,75] were used. The chosen field of study was asked via CATI (open-ended question).

Study success: The instrument to measure the intention to leave consisted of three items on a five-point Likert scale (1 = “fully disagree” to 5 = “fully agree”; an example item (translated) is *“I have often thought about stopping my current study”*). Since only a small number of students dropped out of their latest field of study, realized intentions were not considered. UGPAs were self-reported by the students. The UGPAs in Switzerland range from 6 = “very good” to 4 = “satisfactory”. A UGPA of 3, 2 or 1 is rated as “insufficient” or “fail”. Because of different points of student entry into the field of economics, UGPAs can differ in three ways; they can represent 1) the current UGPA (if the student was currently studying in this field), 2) the former UGPA (if the student switched courses of study or dropped out) or 3) the final UGPA (if the student already graduated). Social integration was measured by using three items on a five-point Likert scale (1 = “fully disagree” to 5 = “fully agree”) [69]; an example item (translated) is *“I have many good friends among my fellow students”*. All scales showed good reliabilities. The reliability of UGPA might be limited because it was self-reported by students, and there were different types of UGPA (final grade vs. average grade).

Control variables: Different variables functioned as control variables. First, general academic skills (mathematics, verbal and cognitive abilities) were considered. To measure them, subtests of a nationwide evaluation of educational reforms of the Abitur (the national school-leaving exam and university entrance qualification; in Switzerland, it is Matura) were used [66]. In addition, the “KFT 4–12 + R” Test by Heller and Perleth [66] was used to test students’ cognitive abilities. The three tests were implemented in a multi-matrix booklet design. Second, the average school grades at the end of upper secondary school were considered. It is important to note that the Swiss grading system ranges from 6 = “very good” to 1 = “fail”. Therefore, higher school grades represent better school performance. Since the latest field of study was focused on, the following academic characteristics of students were controlled for: (1) Prior dropout or change in study course (“prior status”) was operationalized by the final status of the first field of study that students had chosen after graduating from upper secondary school. Therefore, a dichotomous variable was calculated (0 = “retained/finished”, 1 = “dropped out/changed”). Information on (2) the students’ number of semesters completed and (3) the students’ study program (0 = “bachelor’s, 1 = “master’s) was collected. Finally, data on background variables such as gender (0 = female, 1 = male), the Highest International Socio-Economic Index of Occupational Status (HISEI), perceived social support from family (five-point Likert scale; an example item is “*My family really tries to help me”*) [68], school type (0 = BS, 1 = FVBS) and the advanced course in upper secondary school (0 = non-economic, 1 = economic) were considered.

### Missing values

There were missing values (item non-response) for cognitive abilities, mathematics and verbal skills and the further facets of economic competence for 23 to 30 percent of the responses. Therefore, missing values were imputed by multilevel multiple imputation using chained equations (with mice.2l.pan function in the R package *mice*) [76]. Within the imputation model, school classes functioned as a cluster variable, and fixed and random effects, the class mean effects of individual cognitive variables and the individual and stratum-specific weights were considered within the equations. At least ten predictors per outcome variable were used to generate twenty values for each missing value [76,77].

### Analyses

**Fig 2:**
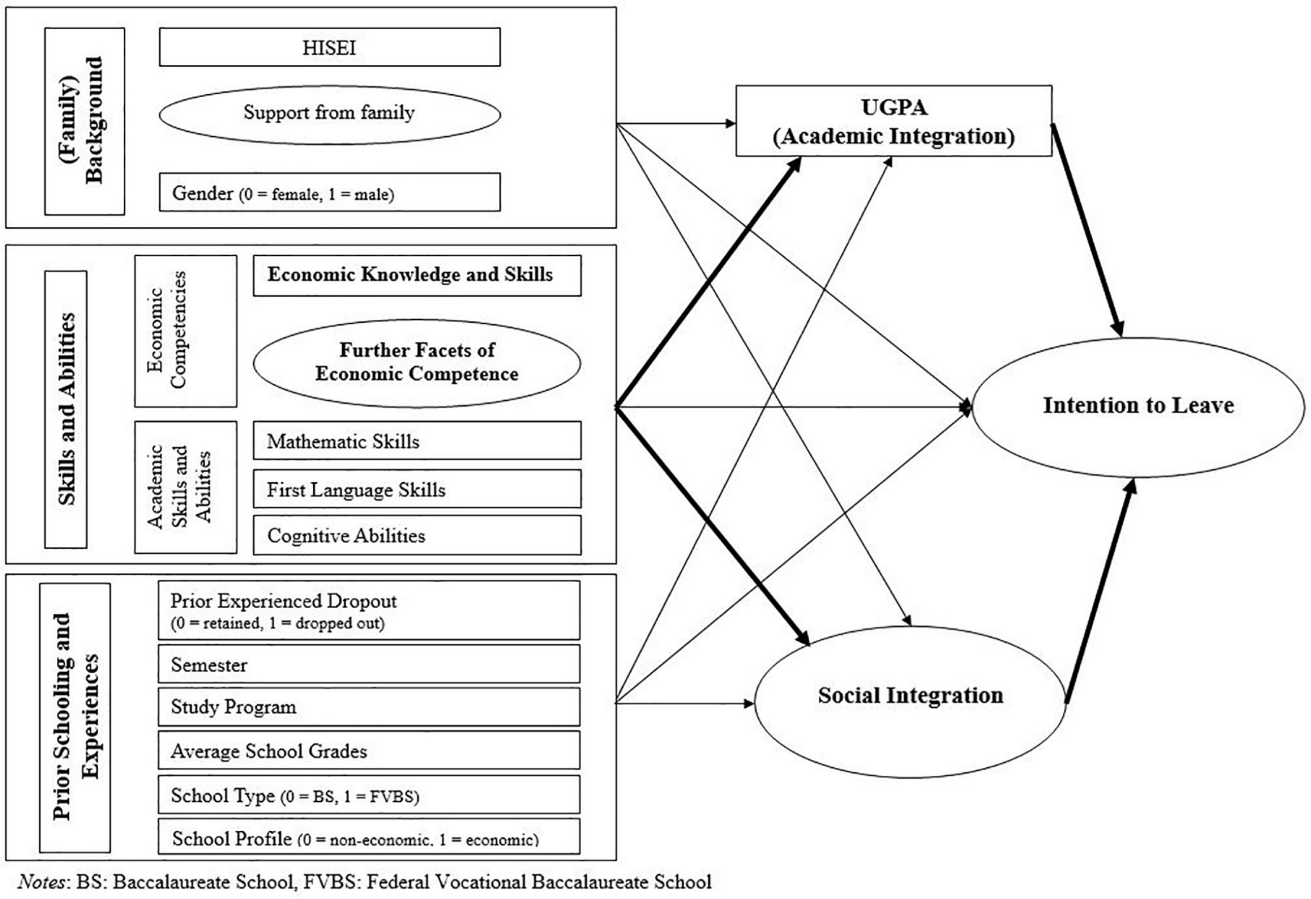
Empirical model to test the effects of economic competencies on the intention to leave economics studies at university. BS = Baccalaureate School, FVBS = Federal Vocational Baccalaureate School.

To estimate individual abilities of economic knowledge and skills, cognitive abilities, and mathematics and verbal skills, mean-weighted likelihood estimators [78] were calculated based on item response theory (IRT) by using the software program “ConQuest” [79] with good reliability (see Table 5 above). Relating to this, a one-dimensional Rasch model [80] was used. In addition to descriptive and bivariate analyses, a structural equation model (SEM) based on the theoretical model (see Fig 2 above) was calculated. Individual background was represented by gender, perceived family support and HISEI. Skills and abilities were represented by economic competencies and general academic skills (cognitive abilities, mathematics and verbal skills). Prior schooling and experiences were represented by school type (BS vs. FVBS), advanced course (Economics vs. Other), average school grades, study program (bachelor’s vs. master’s), semester, and prior dropout experiences. Academic integration (UGPA) and social integration were modelled as mediators. The intention to leave economics was modelled as the dependent variable. Based on explanatory factor analysis (EFA), the four-dimensional structure of the further facets of economic competence (interest, intrinsic motivation, attitude and value-oriented disposition) could not be found in the analyzed sample (see Table 4 above). This lies on strong correlations between the items of these constructs. A common way to methodologically address constructs that are theoretically different but cannot be empirically separated is to use parcels for items that are similar in terms of content [62]. Therefore, the following three parcels were built for the four further facets: (1) all items of interest and intrinsic motivation; (2) 13 items of attitude towards economics (one item of attitude towards economics did not correlate with any other items and was excluded); and (3) 9 items of value-oriented disposition. Finally, these three parcels were used within SEM as indicators for one overlaying dimension. This one-dimensional structure was confirmed by confirmatory factor analysis (CFA) and showed good reliability (see Table 5 above).

### Nested data structure

As the sample was nested in 82 school classes, standard errors were adjusted by using school classes as the cluster variable. School grades and UGPA were standardized by the school class mean and the university mean, respectively. A maximum likelihood estimation with robust standard errors (MLR; sandwich estimator) was used to calculate the standardized estimators within SEM by using MPlus [81–83].

### Model identification, model specification and scaling

Every latent variable was modelled with exactly three indicators (e.g., a just-identified measurement model) [62]. To estimate the mean and variance of the latent variables without over-representing an arbitrary chosen indicator, the effect coding method was used (ibid.). To calculate model fit, the following indices were adopted: chi-square (χ^2^) and degrees of freedom (df); the comparative fit index (CFI); the root mean square error of approximation (RMSEA); and the standardized root mean square residual (SRMR). Since model fit indices have their specific advantages and disadvantages, different indices are used to get a clear picture of the “true” fit. For instance, the χ^2^/df ratio is the basis for many other fit indices and is also the most popular one, but it is often too liberal regarding the normal distribution of variables. CFI represents an incremental measure of fit that pays a penalty for every parameter estimated. Therefore, it considers model complexity. Finally, RMSEA and SRMR represent absolute measures of fit, and SRMR is used because it considers different scales in the variables within the model. However, SRMR does not penalize model complexity. For more details on this topic, see [84]. Against this background, different model fit indices are presented to obtain a better picture of the overall fit.

Since academic and social integration and the intention to leave were measured simultaneously at T2, the causal ordering of these variables cannot be ruled out by different points in time [85]. Because of this, there are several other equivalent models in terms of goodness of fit [86]. In this regard, five equivalent models are tested based on academic and social integration and the intention to leave. In the first model, intention to leave is modelled as the mediator, and academic and social integration are modelled as criterions. In the second and third model, academic and social integration are modelled as single mediators. In the fourth and fifth model, academic and social integration, respectively, is considered as the only criterion. These additional models are discussed in the results.

Additionally, several nested models are tested to more strictly prove the theoretical model. First, it is assumed that the variables of the preceding block [86] are fully mediated by academic and social integration (see Fig 1). Second, it is tested whether academic and social integration are uncorrelated. These nested models are tested against one another. Regarding the preceding block, it is assumed that all variables are correlated and that “they are not attempting to impose causal order” [86, p. 257, 87].

### Ethics statement

All relevant national standards concerning the recruitment and information of the participating adolescents, schools, and organizations were respected (with written informed consent). Participation in this study was voluntary. At the first point of measurement, the school administrations approved all surveys consistent with the Swiss standards for school surveys at the time. In addition, all participants were older than 16, and most of them were already of legal age. Since our research project is not associated with any risks or burdens, no additional consent was required from legal representatives (Art. 24,1 HFG). For the second measurement date, all Swiss standards for data collection were also complied with. All participants were of legal age, fully informed and agreed to participate.

## Results

### Descriptive statistics and interrelations

Table 6 gives an overview of the means and standard deviations of the modelled variables. These findings indicate that economics students were above average in all facets of economic competencies but below average in mathematics and verbal skills. In addition, over 60 percent were male students, which is consistent with federal statistical data [88]. Finally, economics students had relatively high UGPA, high social integration and a low intention to leave, with relatively low standard deviations. The bivariate correlations between the independent variables were generally small or moderate (see Table 7). Regarding economic knowledge and skills, there were moderate positive correlations with the further facets of economic competence and average school grades. In addition, FVB students showed lower mathematics skills than BS students and were more often enrolled in an advanced economics course. Students who were enrolled in a master’s program showed higher scores in cognitive abilities, mathematics and verbal skills than students who were enrolled in a bachelor’s program. Finally, the semester and the study program were correlated because bachelor’s programs (generally 6 semesters) are longer than master’s programs (generally 4 semesters).

**Table 6:** Means and standard deviations of the variables (weighted).

Variable	Mean	Standard Deviation
***Independent Variables***		
Economic Knowledge and Skills^a^	0.45	1.06
Further Facets of Economic Competence^a^^,^^c^	0.34	0.70
Mathematics Skills^a^	-0.22	1.03
Verbal Skills^a^	-0.25	1.17
Cognitive Abilities^a^	-0.05	1.05
Average School Grades^b^	4.65	0.40
Family Support^c^	4.31	0.78
Gender (0 = female, 1 = male)	0.63	0.48
School Type (0 = BS, 1 = FVBS)	0.44	0.49
Advanced Course (0 = Non-Economic, 1 = Economic)	0.51	0.50
Prior Status (0 = remain, 1 = drop-out/change)	0.22	0.41
Study Program (0 = Bachelor’s, 1 = Master’s)	0.20	0.40
Semester	4.30	2.41
HISEI^d^	64.5	16.4
***Mediators***		
Academic Integration (GPA/Final Grade)^b^	4.90	0.36
Social Integration^c^	3.73	0.70
***Dependent Variable***		
Intention to Leave^c^	1.47	0.67

^a^Values were z-standardized (mean = 0.0, SD = 1.0) in the overall sample (N = 520). Means above 0.0 indicate above-average performance, and means lower than 0.0 indicate under-average performance.

^b^Grades range from 6 (very good) to 1 (insufficient).

^c^Means and standard deviations were estimated based on CFA. These scales range from 5 (fully agree) to 1 (fully disagree).

^d^HISEI ranges from 0 to 100 points.

**Table 7:** Correlations between independent variables.

	(1)	(2)	(3)	(4)	(5)	(6)	(7)	(8)	(9)	(10)	(11)	(12)	(13)	(14)
Ec. Knowledge and Skills (1)	1	.46**	-.05	.19*	.15	.37**	-.24*	.28**	.05	.18	-.23*	.13	-.01	-.06
Further Facets of Economic Competence (2)		1	.04	.05	.05	.23	-.06	.24*	-.10	.20	-.09	.14	-.02	-.02
Mathematics Skills (3)			1	.19	.59**	.04	.09	.17	-.72**	-.42**	.09	.38**	-.17	.05
Verbal Skills (4)				1	.19	.13	.06	-.06	-.11	-.05	-.05	.22**	-.16*	-.04
Cognitive Abilities (5)					1	.15	-.12	.23*	-.21	-.21	-.02	.18*	-.04	.00
Average School Grades (6)						1	-.12	-.19	.05	.11	-.10	.25*	.04	-.02
Family Support (7)							1	-.13	.03	.10	.05	-.11	-.09	.06
Gender (0 = female, 1 = male) (8)								1	-.08	.11	.13	-.06	.08	.04
School Type (0 = BS, 1 = FVBS) (9)									1	.51**	.05	-.40**	.19	-.17
Advanced Course (0 = Non-Ec., 1 = Ec.) (10)										1	.11	-.24**	.20*	-.12
Prior Status (0 = remain, 1 = drop out) (11)											1	-.19	-.30**	-.08
Study Program (0 = Bachelor’s, 1 = Master’s) (12)												1	-.55**	.09
Semester (13)													1	.04
HISEI (14)														1

*p < .05

**p < .01.

Table 8 shows the bivariate correlations between the exogenous and endogenous variables. Economic knowledge and skills were positively correlated with UGPA. There were no interrelations between the intention to leave and the facets of economic competencies. Students with better school grades, male students, and students with economics as their advanced course showed a higher UGPA. Considering social integration, students who perceived higher family support and had remained in their first field of study were more integrated. There was a moderate negative correlation between students’ cognitive abilities and their intention to leave. FVB students showed a higher intention to leave than BS students. Finally, students who were more integrated in both the academic and social environment had a lower intention to leave. The residuals of academic and social integration were uncorrelated.

**Table 8:** Correlations between the exogenous and endogenous variables.

ExogenousVariables	Endogenous Variables	UGPA (academic integration)	Social integration	Intention to Leave
Ec. Knowledge and Skills		.36**	-.03	.03
Further Facets of Economic Competence		-.10	-.03	-.01
Mathematics Skills		-.08	-.07	-.14
Verbal Skills		.08	-.05	.09
Cognitive Abilities		.07	.07	-.30*
Average School Grades		.43**	-.14	.11
Family Support		-.03	.28*	-.07
Gender (0 = female, 1 = male)		-.23*	-.06	-.14
School Type (0 = BS, 1 = FVBS)		-.09	-.17	.24**
Advanced Course (0 = Non-Ec., 1 = Ec.)		.21*	.07	-.16
Prior Experienced Dropout (0 = remained, 1 = dropped out)		-.13	-.34**	.37**
Study Program (0 = Bachelor’s, 1 = Master’s)		.23	-.05	.17
Semester		< .01	.10	.24
HISEI		.07	.05	-.26**
UGPA (Academic Integration)		1	.08^a^	-.36*
Social Integration		--	1	-.53**

Ec. = Economic

^a^Residual correlation

*p<0.05

**p<0.01

### Prediction of the intention to leave economics

Fig 2 shows the empirical model that was tested (original model).

**Fig 3:**
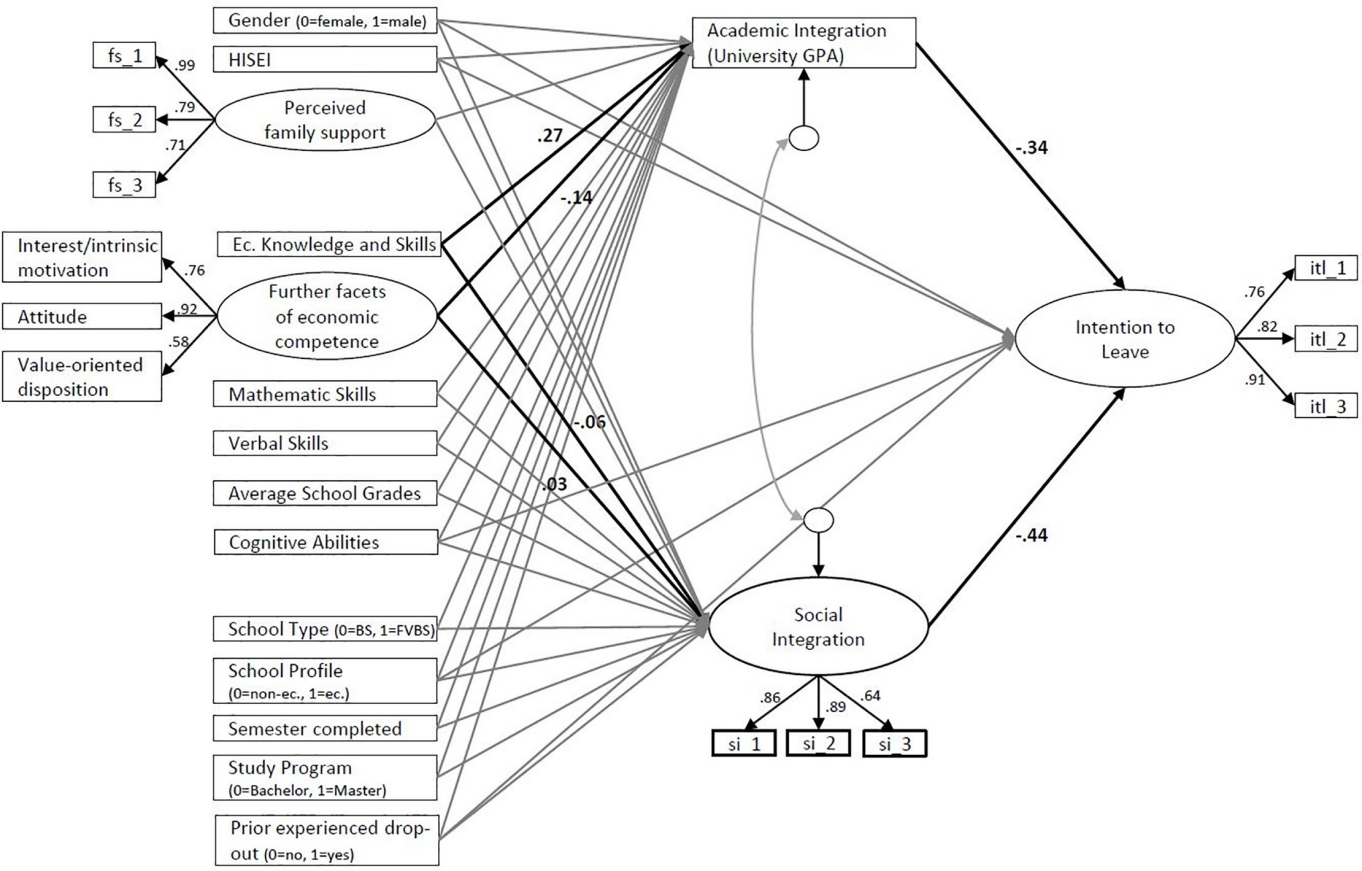
Path diagram regarding the effects of economic competencies on the intention to leave economics studies at university. Ec. = Economics, GPA = Grade Point Average, UGPA = University Grade Point Average, BS = Baccalaureate School, FVBS = Federal Vocational Baccalaureate School.

Fig 3 displays the complete SEM based on the theoretical model. The symbolism in the path diagram follows the McArdle-McDonald reticular action model (RAM) [89]. For more information on RAM, see [90]. Due to the number of path coefficients, just the path coefficients of economic competencies are shown in the path diagram. The results are reported in Tables 9 and 10. The “preceding” block in the path diagram is assumed to be fully correlated [86]. Next, equivalent models were tested to prove competing hypotheses and to support the assumed mediation as described in the original model. The reasons for testing these models are given in the model identification chapter. The overall results of the testing of the original and the equivalent models are summarized in S1 Table. The only model that represents meaningful results regarding the theoretical considerations based on the path coefficients (direct and indirect effects) is the original model [91]. Against this background, it is decided to use the original model.

**Table 9:** Results of SEM to predict the intention to leave economics (direct effects).

Exogenous Variables	Endogenous Variables	Academic Integration (UGPA)	Social Integration	Intention to Leave
***(Family) Background***				
HISEI		.06 (.08)	.05 (.08)	**-.14**^†^ **(.08)**
Perceived Support from Family		-.02 (.10)	**.21**^†^ **(.12)**	.00 (.00)
Gender (0 = Female, 1 = Male)		-.15 (.12)	-.05 (.14)	-.11 (.10)
***Skills and Abilities***				
Economic Knowledge and Skills		**.27**^†^ **(.15)**	-.06 (.18)	.00 (.00)
Further Facets of Economic Competence		-.14 (.16)	.03 (.19)	.00 (.00)
Mathematics Skills		-.23 (.18)	-.15 (.21)	.00 (.00)
Verbal Skills		-.03 (.07)	.01 (.10)	.00 (.00)
Cognitive Abilities		-.04 (.15)	.09 (.18)	-.27* (.11)
***Prior Schooling and Experiences***				
Prior Experienced Dropout (0 = retained, 1 = dropped out)		-.29* (.12)	-.46** (.13)	.00 (.00)
Study Program (0 = Bachelor’s, 1 = Master’s)		.04 (.12)	-.27 (.18)	-.20* (.09)
Semester		-.12 (.12)	-.09 (.17)	-.22* (.09)
Average School Grades		.30* (.13)	-.08 (.16)	.33** (.10)
Advanced Course (0 = non-economic, 1 = economic)		-.04 (.11)	.02 (.15)	-.12 (.08)
School Type (0 = BS, 1 = FVBS)		**-.25**^†^ **(.15)**	**-.28**^†^ **(.15)**	.00 (.00)
***Mediators***				
Academic Integration (UGPA)		--	--	-.34** (.16)
Social Integration		--	--	-.44** (.09)
*Adjusted R-Square*		.*39*	.*29*	.*56*

*Model fit information*: χ^2^ = 212, df = 160, CFI = 0.926, RMSEA = 0.048, SRMR = 0.050

**p<0.01

*p<0.05

^†^p<0.10; significant results are highlighted in bold

HISEI: Highest International Socio-Economic Index of Occupational Status (by family), BS: Baccalaureate School, FVBS: Federal Vocational Baccalaureate School, UGPA: university grade point average

**Table 10:** Indirect and total effects on the intention to leave.

Independent Variable	Indirect Effect (UGPA)	Indirect Effect (Social Integration)	Total Indirect Effect	Total Effect
Economic Knowledge and Skills	**-.09**^†^ **(.05)**	.03 (.08)	-.06 (.11)	-.06 (.11)
Average School Grades	-.10 (.07)	.04 (.07)	-.07 (.11)	.26 (.16)
Cognitive Abilities	.02 (.05)	-.04 (.08)	-.02 (.11)	-.29* (.14)
Prior Experienced Dropout (0 = retained, 1 = dropped out)	.10 (.07)	.20** (.07)	.30** (.10)	.30** (.10)
School Type (0 = BS, 1 = FVBS)	.09 (.08)	.12 (.08)	**.21**^†^ **(.11)**	.21* (.11)
Perceived Support from Family	.01 (.03)	**-.09**^†^ **(.05)**	-.09 (.07)	-.09 (.07)
HISEI	-.02 (.03)	-.02 (.04)	-.04 (.04)	-.18* (.09)
Semester	.04 (.04)	.04 (.07)	.08 (.09)	-.14 (.14)
Study Program (0 = Bachelor’s, 1 = Master’s)	-.01 (.04)	.12 (.08)	.11 (.09)	-.10 (.13)

Significant results are highlighted in bold

BS: Baccalaureate School, FVBS: Federal Vocational Baccalaureate School

**p<0.01

*p<0.05

^†^p<0.10

significant results are highlighted in bold

In the following steps, nested models were examined. In the first step, the direct effects of economic competencies on the intention to leave were constrained to be zero to test for total mediation (c.f. Fig 1). These constraints led to a small deterioration in model fit (ΔCFI = 0.001) [84]. In the same way, further direct effects on the intention to leave (further skills and abilities, prior schooling and experiences and (family) background) were constrained to be zero. With the exception of cognitive abilities, average school grade, school profile, SES, gender, study program and semester, these constraints led to a small improvement in model fit (ΔCFI = 0.002). In addition, the correlation between academic and social integration was constrained to be zero, which also led to a small improvement in model fit (ΔCFI = 0.002). In addition, a nested model was tested in which the indirect effect of economic knowledge and skills on the intention to leave (mediated by academic integration) was constrained to be zero. This led to a significant deterioration in model fit (ΔCFI = 0.005). A similar outcome occurred with a model where all direct effects were constrained to be zero. Table 11 summarizes the nested models. The statistical differences among the models were further tested by using Chi-Square difference tests. As a result, it was decided to use the more restrictive model (model 4 in Table 11), which still shows an appropriate model fit. For more details on the selection of the nested models, see [84].

**Table 11:** Test of different models nested in the original model.

Model	Constraints (cumulative)	χ^2^	df	RMSEA	CFI	SRMR
1	None (original model)	205.7	152	.050	.923	.049
2	Total mediation of economic competencies	208.7	154	.050	.922	.050
3	Total mediation of further variables^1^	212.3	159	.048	.924	.050
4	Zero correlation between academic and social integration	212.0	160	.048	.926	.050
5	No mediation of economic knowledge and skills by academic integration	216.2	161	.049	.921	.050
6	Total mediation of all variables	247.0	166	.059	.884	.063

There is no significant difference in the model fit between the nested models based on Chi-Square difference tests, except for models 5 (p<0.05) and 6 (p<0.01).

^1^The following variables are included: family support; prior experienced drop-out; economic competencies; mathematic and verbal skills; and school type. Cognitive abilities, school grades, school profile, gender, SES, study program and semester led to deterioration and are excluded.

Both academic and social integration were strong predictors of the intention to leave and were mediating effects of students’ individual characteristics in different ways. Regarding academic integration, students with higher economic knowledge and skills, students with better school grades, BS students and students who did not experience a prior dropout also showed higher UGPAs. Additionally, students who perceived better support from their families, BS students and students who did not experience a prior dropout were better socially integrated. Finally, students with higher cognitive abilities at the end of upper secondary school, students with a higher SES, students of higher semesters, and students who were enrolled in a Master’s program had a lower intention to leave.

Table 10 shows the results of the (specific) indirect and total effects. The effect sizes were generally small or moderate. Economic knowledge and skills was a strong predictor of UGPA, but there was no positive direct effect on the intention to leave. However, there was also a negative indirect effect on the intention to leave that was mediated by UGPA. Following the latest approaches [92,93], mediating effects might also appear if the total effect is not significant. The main reason for this occurrence is that the predictors show no total effect due to adverse (indirect and direct) effects. Therefore, bootstrapping with 2,000 random samples was performed to test whether zero could be a plausible value of the indirect effect of economic knowledge and skills on the intention to leave (mediated by UGPA) [94]. The results showed that the 95% confidence interval (CI) did not include zero as a plausible value. Against this background, the first hypotheses can be accepted. However, the second hypothesis must be rejected because the further facets of economic competence had no effect on the intention to leave.

In addition to economic competencies, further indirect effects can be identified based on Table 10. First, prior dropout experiences had a strong negative total effect on the intention to leave, which was partially mediated by social integration. Second, BS students had a lower intention to leave. Here, only the sum of the indirect effects became significant. Finally, although students’ perceived support from family showed no significant total effect on the intention to leave, it had a small indirect effect that was mediated by students’ social integration. Bootstrapping revealed that the 95% CI did not include zero as a plausible value. Therefore, the mediating effect of perceived family support on social integration was confirmed. Regarding the semester, study program and HISEI, no indirect effects could be found. Finally, one unexpected result, namely, the positive direct effect of school grades on the intention to leave, was further investigated by using bootstrapping. The result showed that zero was a plausible value on the 95% CI for this effect size.

## Conclusion, limitations and implications

Considering the importance of an economic education at the upper secondary level, the major concern regarding student retention within higher education and the worldwide popularity of studying economics, the present study examined the effects of economic competencies at the end of upper secondary school on the intention to drop out of economic studies at universities by using a longitudinal and representative sample of Swiss students.

Economic knowledge and skills at the end of upper secondary school had a moderate effect on UGPA in economics and a small indirect effect on the intention to leave, which was mediated by UGPA. Two findings were unexpected. First, there were no effects of the further facets of economic competence which is not consistent with previous findings [56]. One reason for this finding could be the operationalization of these constructs. The items mostly referred to the subject “Economics and Law”, which students in the sample had chosen as a basic or advanced course. Students’ interests, motivation, attitude or value-oriented disposition regarding a school subject may only partly reflect their psychological disposition in relation to the domain of “economics”. Second, there were either no direct effects or positive direct effects of economic knowledge and skills and school grades on the intention to leave. However, by using bootstrapping, the results regarding the 95% CI showed that zero was a plausible value. Therefore, these results should not be overrated.

In addition to the effects of economic competencies, further effects are relevant to discuss. First, students who had studied economics but had experienced a previous dropout were less academically and socially integrated. The possible reasons for this finding are that (1) these students probably had a lower general ability to study and therefore were less academically integrated and that (2) the students joined a cohort of people who they did not know, which led to lower social integration. Second, BS students showed a lower intention to leave. This is probably because FVB students have also completed a vocational education and training program that enables them to apply for a job in their profession. Therefore, opportunity costs for studying are probably higher for FVB students than for BS students. Third, male students showed a lower intention to leave. Although this effect is small and becomes not significant, this might be because of the overall superiority of male students regarding economic competencies that has been observed in various countries [48,95,96]. Fourth, students with a lower SES showed a stronger intention to leave, which is consistent with other studies. The reasons for this effect can be seen, for example, in the lower social, economic and cultural capital that these students have at their disposal [97,98] or the differences in the cost-benefit-calculations regarding the continuation of studying [99, 100]. Finally, the results show that when the study in economics is more advanced, the intention to leave is lower. This is probably due to rising opportunity costs as studies progress.

In general, the theoretical model (see Fig 1) can be represented well by the data (see Tables 9 and 11). Over 55% of the variance in the students’ intention to drop out can be explained. Considering the relatively long period of time between T1 and T2 and the focus on the latest field of study, the effect sizes should not be underrated. Against this background, the extent to which the effects of students’ economic competencies at the end of upper secondary school were predictive is remarkable. In addition, it must be emphasized that the effects of economic competencies were controlled for typical predictors, such as academic abilities (mathematics skills, verbal skills, and cognitive abilities), gender, SES, and prior schooling (school grades, type of school, and advanced course).

Some limitations and research desiderata must be noted. First, from a methodological point of view, the most critical point must be seen in the simultaneous measurement of UGPA, social integration and intention to leave. Although competing hypotheses could be rejected, the assumed causality regarding mediation must be interpreted with caution due to missing a third point of measurement. As a result, the mediating effects should better be interpreted as indirect effects without causal meaning. Second, the empirical model did not include all aspects that are formulated [26,27]. The most crucial point is that the initial commitments before entering university were not measured. The same is true for subsequent commitments. Future research should include more measurement points to reflect the entire process of dropout decisions in higher education. Third, although it was most valid to ask for students’ intention to leave concerning their latest study, the effects were probably biased by students’ prior experiences and the fact that students who were not currently studying (e.g., because they had already finished or dropped out) had to be asked questions retrospectively. To address this, prior dropout experiences, the number of semesters completed and the study programs were controlled. However, because of the sample size, it was not possible to model these variables as additional moderators. Therefore, future research should focus on the first study choice to (1) measure initial and subsequent commitments and (2) prevent retrospectivity. In addition, realized dropout decisions should be included. Fourth, considering the review of the literature on student retention, variables such as students’ personality traits, metacognitive strategies, and PSFs (e.g., academic self-efficacy) should be considered as further individual characteristics. Fifth, again, due to the sample size, different courses within economics were not separated; that is students in (political) economics, business and administration, economics education, etc. were put together in one group. However, differences in these courses in terms of requirements and dropout rates and differences across institutions must be assumed. Finally, since this study focuses on Swiss students, further investigations on this topic are strongly recommended to replicate these findings, particularly due to the system-related characteristics of the Swiss education system (e.g., basic education in economics at the upper secondary level).

Regardless of these limitations and open questions, this study is the first to present the longitudinal effects of economic competencies at the end of upper secondary school on study success in economic studies, and it supports the importance of fostering economic knowledge and skills through secondary education. This study helps fill the gap in the research regarding the observation of domain-specific effects on study success. By focusing on students of economics, the study addresses a broad field within higher education, and the results are relevant for many countries worldwide. A major point is that this study did not use a typical study ability test (e.g., SAT/ACT or GRE) to predict academic performance [34]. The focus was on the general skills and abilities that are necessary to act as responsible citizens within complex economic societies. Against this background, the results strongly support the assumption that fostering economic competencies at the secondary level will not only improve students’ ability to act as informed citizens in modern societies and to use this competence for their personal financial behavior, which has been most discussed in the past [20, 46, 51, 59], but also improves students’ domain-specific abilities to study in the broad field of economics. In this regard, students with higher economic competencies benefit in that they perform better in economics and are less likely to intend to drop out. Therefore, it can be assumed that universities will also benefit from lower dropout and turnover rates in this field. In addition, it must be noted that economics is the field with the highest turnover in Switzerland. Against this background, the findings underline the importance of the general skills and abilities in terms of economic literacy that go beyond simple improvements in personal finances or an understanding of economic principles.

## Supporting information

S1 Table Summary of the test results of equivalent models regarding the endogenous variables. (DOCX)

S1 File Tables and path diagrams of the original and equivalent models. (ZIP)

S1 Raw Data Raw data on economic and further competencies and study success of Swiss students. (XLSX)
