# Supplementary material for: Predicting economics student retention in higher education: The effects of students’ economic competencies at the end of upper secondary school on their intention to leave their studies in economics
Source: PLoS One. 2020 Feb 5;15(2):e0228505. doi: 10.1371/journal.pone.0228505 (PMC7001938; doi:10.1371/journal.pone.0228505)
Supplement: S1 Table — (DOCX) [file pone.0228505.s001.docx]

**S1 Table. Summary of the test results of equivalent models regarding the endogenous variables**

| Model | Characteristics | Result |
| --- | --- | --- |
| 1 | **Original model**  Mediators: Academic and social integration  Dependent variable: Intention to leave  Hypotheses: Indirect effect of economic competencies on intention to leave mediated by academic integration. | Academic and social integration function as mediators. Economic knowledge and skills and school grades are mediated by academic integration. Prior experienced drop-out and family support are mediated by social integration.  Hypothesis can be confirmed regarding economic knowledge and skills. |
| 2 | **Equivalent model 1**  Mediator: Intention to leave  Dependent Variables: Academic and social integration  Competing hypothesis: Indirect effect of economic competencies on academic and/or social integration mediated by the intention to leave. | Intention to leave functions as a mediator but only for socioeconomic status and prior-experienced drop-out. No mediation of students’ skills and abilities (including economic competencies). Primarily direct effects on academic and social integration. No meaningful results from a theoretical perspective.  Competing hypothesis must be rejected. |
| 3 | **Equivalent model 2**  Mediators: Academic integration and intention to leave  Dependent variable: Social integration  Competing hypothesis: Indirect effect of economic competencies on social integration mediated by the intention to leave and/or academic integration. | No mediation by academic integration. Small indirect effects of socioeconomic background and prior experienced drop-out on social integration mediated by the intention to leave. No mediation of students’ cognitive dispositions.  Competing hypothesis must be rejected. |
| 4 | **Equivalent model 3**  Mediators: Social integration and intention to leave  Dependent variable: Academic integration  Competing hypothesis: Indirect effect of economic competencies on social integration mediated by the intention to leave and/or academic integration. | No mediation by social integration. Small indirect effects of socioeconomic background and prior experienced drop-out on academic integration mediated by the intention to leave. No mediation of students’ cognitive dispositions.  Competing hypothesis must be rejected. |
| 5 | **Equivalent model 4**  Mediator: Social integration  Dependent variables: Academic integration and intention to leave  Competing hypothesis: Indirect effect of economic competencies on academic integration and/or intention to leave (mediated by social integration). | Mediation of prior experienced dropout and family support regarding intention to leave (see the original model). No mediation concerning academic integration. No mediation of students’ cognitive dispositions.  Competing hypothesis must be rejected. |
| 6 | **Equivalent model 5**  Mediator: Academic integration  Dependent variables: Social integration and intention to leave  Competing hypothesis: Indirect effect of economic competencies on social integration (mediated by academic integration) | Mediation of economic competencies and school grades regarding intention to leave (see the original model). No mediation concerning social integration.  Competing hypothesis must be rejected. |
