# Supplementary material for: Predicting economics student retention in higher education: The effects of students’ economic competencies at the end of upper secondary school on their intention to leave their studies in economics
Source: PLoS One. 2020 Feb 5;15(2):e0228505. doi: 10.1371/journal.pone.0228505 (PMC7001938; doi:10.1371/journal.pone.0228505)
Supplement: S1 File — (ZIP) [file pone.0228505.s002.zip › S1 Figure_Additional path diagrams.pdf]

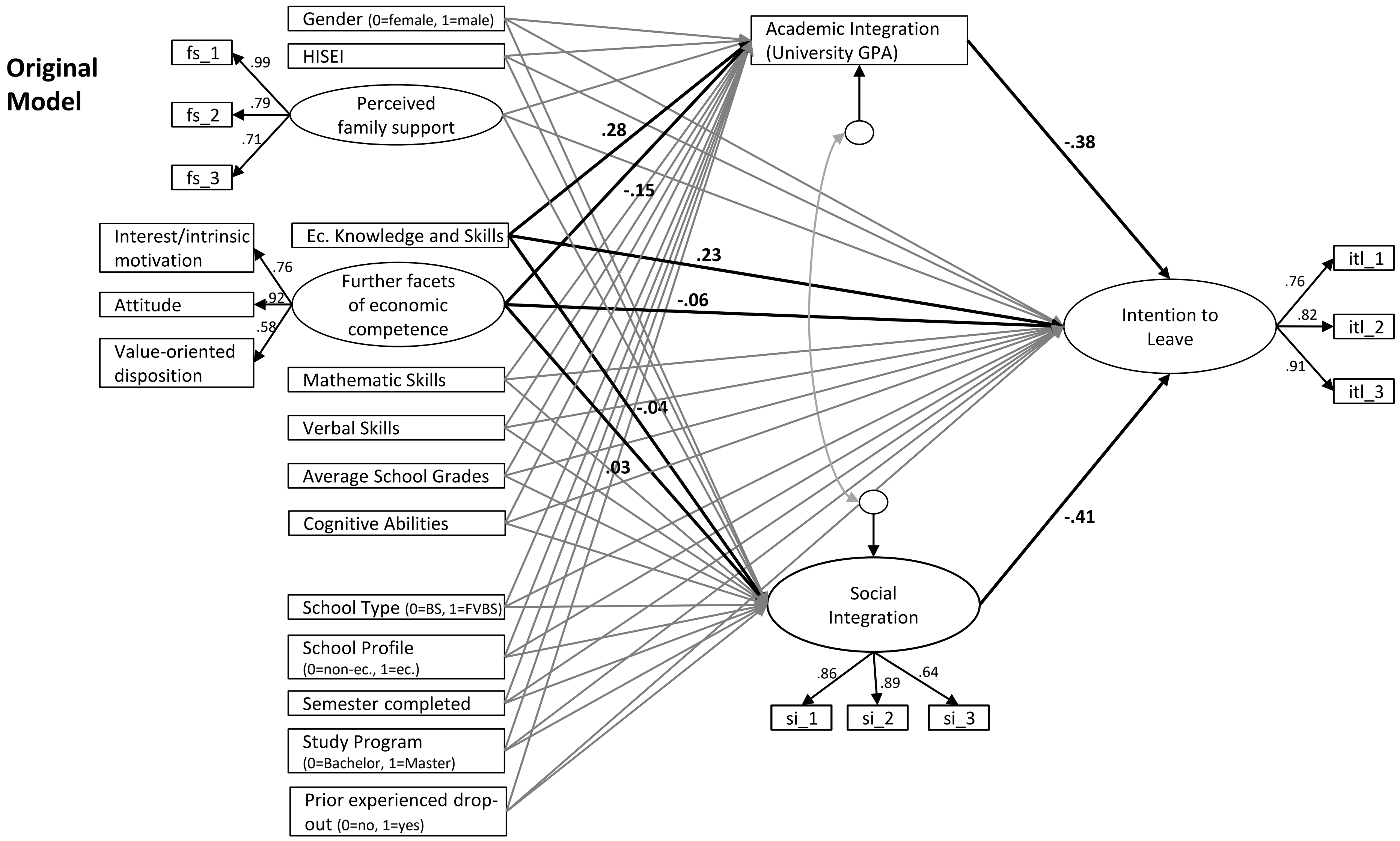

Original Model
Gender (0=female, 1=male)
HISEI
Perceived family support
fs_1
fs_2
fs_3
.99
.79
.71
Ec. Knowledge and Skills
Interest/intrinsic motivation
Attitude
Value-oriented disposition
.76
.92
.58
Further facets of economic competence
Mathematic Skills
Verbal Skills
Average School Grades
Cognitive Abilities
School Type (0=BS, 1=FVBS)
School Profile (0=non-ec., 1=ec.)
Semester completed
Study Program (0=Bachelor, 1=Master)
Prior experienced drop-out (0=no, 1=yes)
Academic Integration (University GPA)
.28
-.15
.23
-.06
-.04
.03
-.38
-.41
Intention to Leave
itl_1
itl_2
itl_3
.76
.82
.91
Social Integration
si_1
si_2
si_3
.86
.89
.64

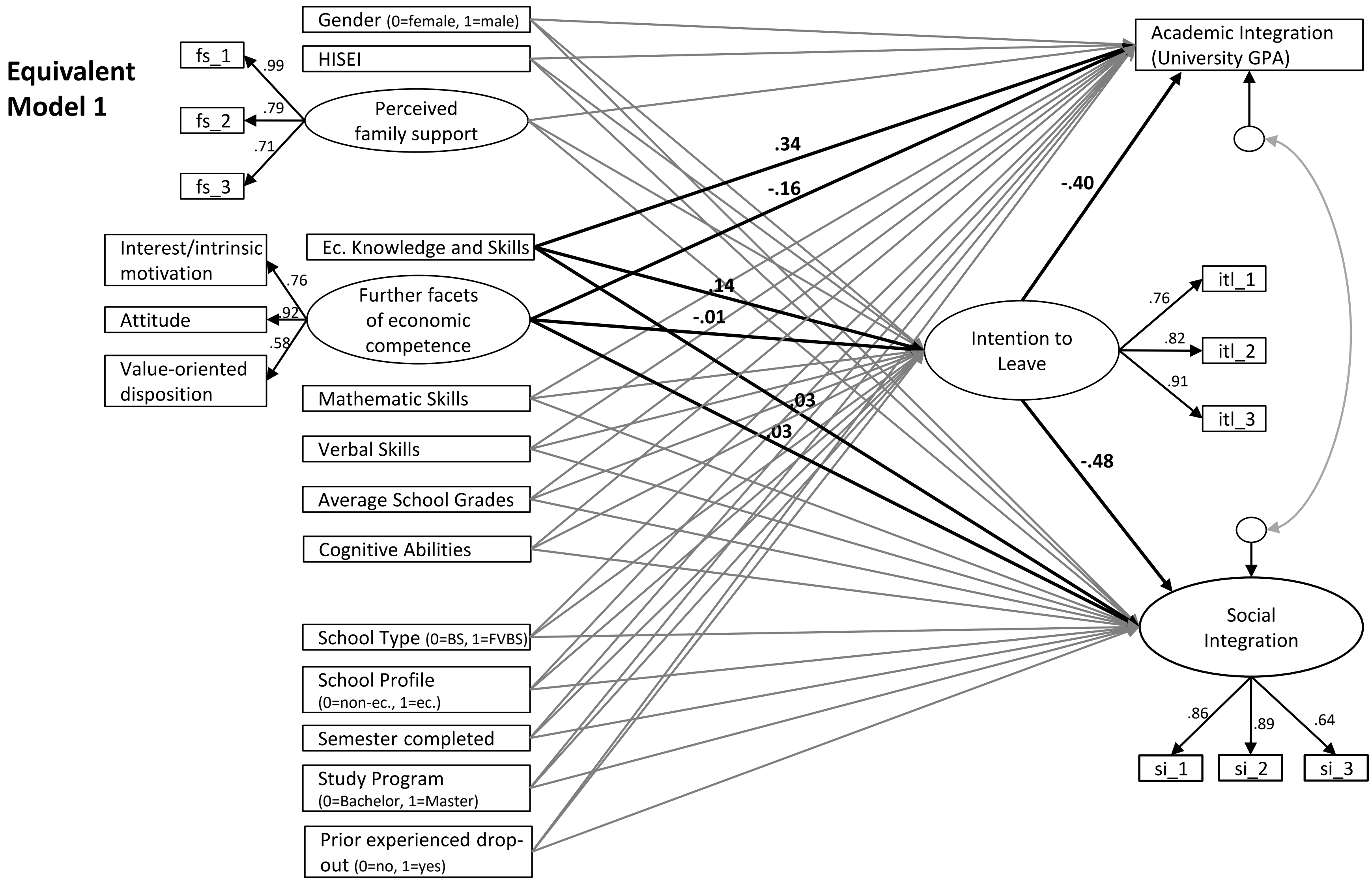

Equivalent Model 1
Gender (0=female, 1=male)
HISEI
fs_1
fs_2
fs_3
.99
.79
.71
Perceived family support
Ec. Knowledge and Skills
Interest/intrinsic motivation
Attitude
Value-oriented disposition
.76
.92
.58
Further facets of economic competence
Mathematic Skills
Verbal Skills
Average School Grades
Cognitive Abilities
School Type (0=BS, 1=FVBS)
School Profile (0=non-ec., 1=ec.)
Semester completed
Study Program (0=Bachelor, 1=Master)
Prior experienced drop-out (0=no, 1=yes)
.34
-.16
.14
-.01
.03
.03
-.40
-.48
Academic Integration (University GPA)
Intention to Leave
itl_1
itl_2
itl_3
.76
.82
.91
Social Integration
si_1
si_2
si_3
.86
.89
.64

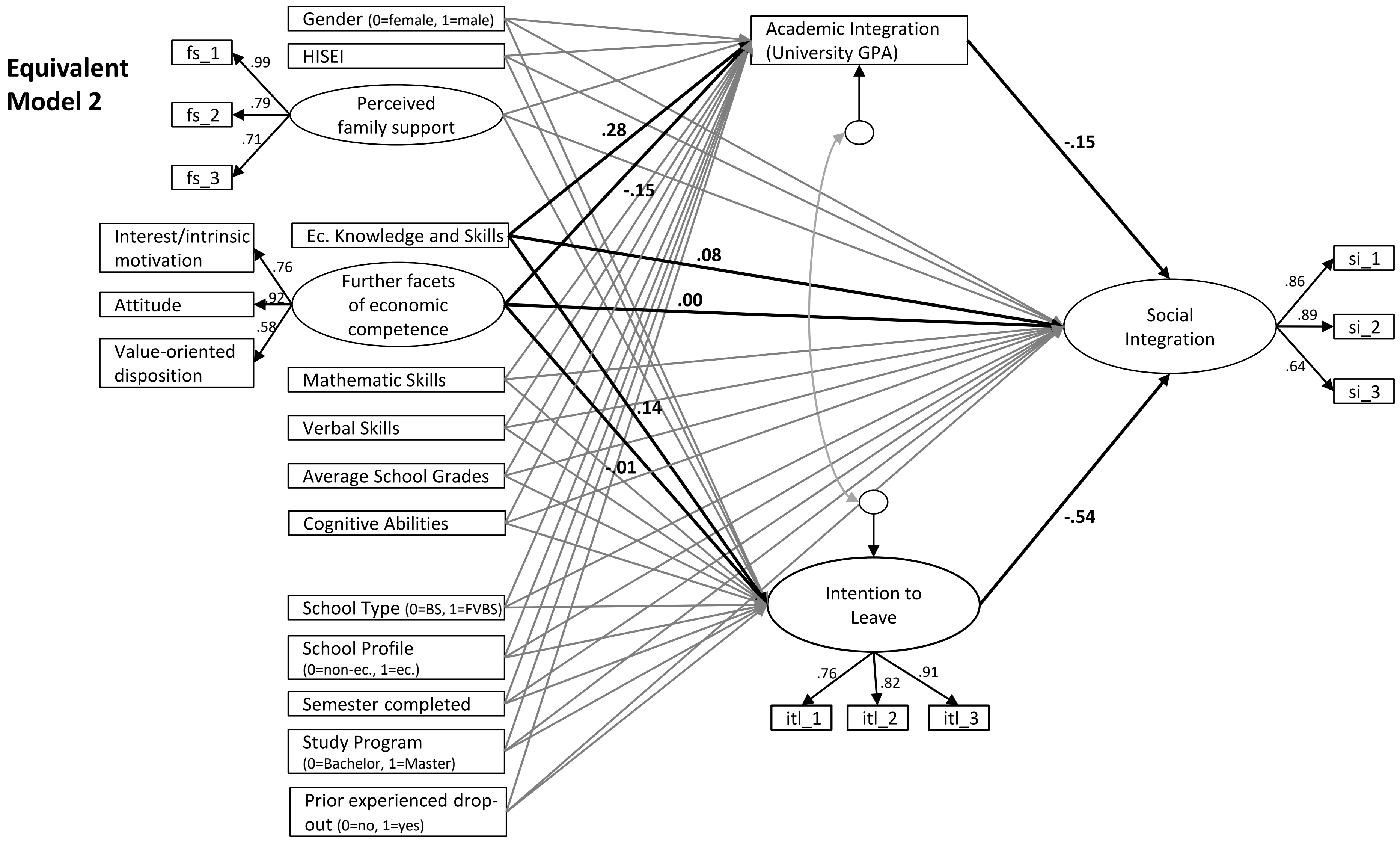

Equivalent Model 2
Gender (0=female, 1=male)
HISEI
Perceived family support
fs_1
fs_2
fs_3
.99
.79
.71
Ec. Knowledge and Skills
Interest/intrinsic motivation
Attitude
Value-oriented disposition
Further facets of economic competence
.76
.92
.58
Mathematic Skills
Verbal Skills
Average School Grades
Cognitive Abilities
School Type (0=BS, 1=FVBS)
School Profile (0=non-ec., 1=ec.)
Semester completed
Study Program (0=Bachelor, 1=Master)
Prior experienced drop-out (0=no, 1=yes)
Academic Integration (University GPA)
.28
-.15
.08
.00
.14
-.01
-.15
-.54
Social Integration
si_1
si_2
si_3
.86
.89
.64
Intention to Leave
itl_1
itl_2
itl_3
.76
.82
.91

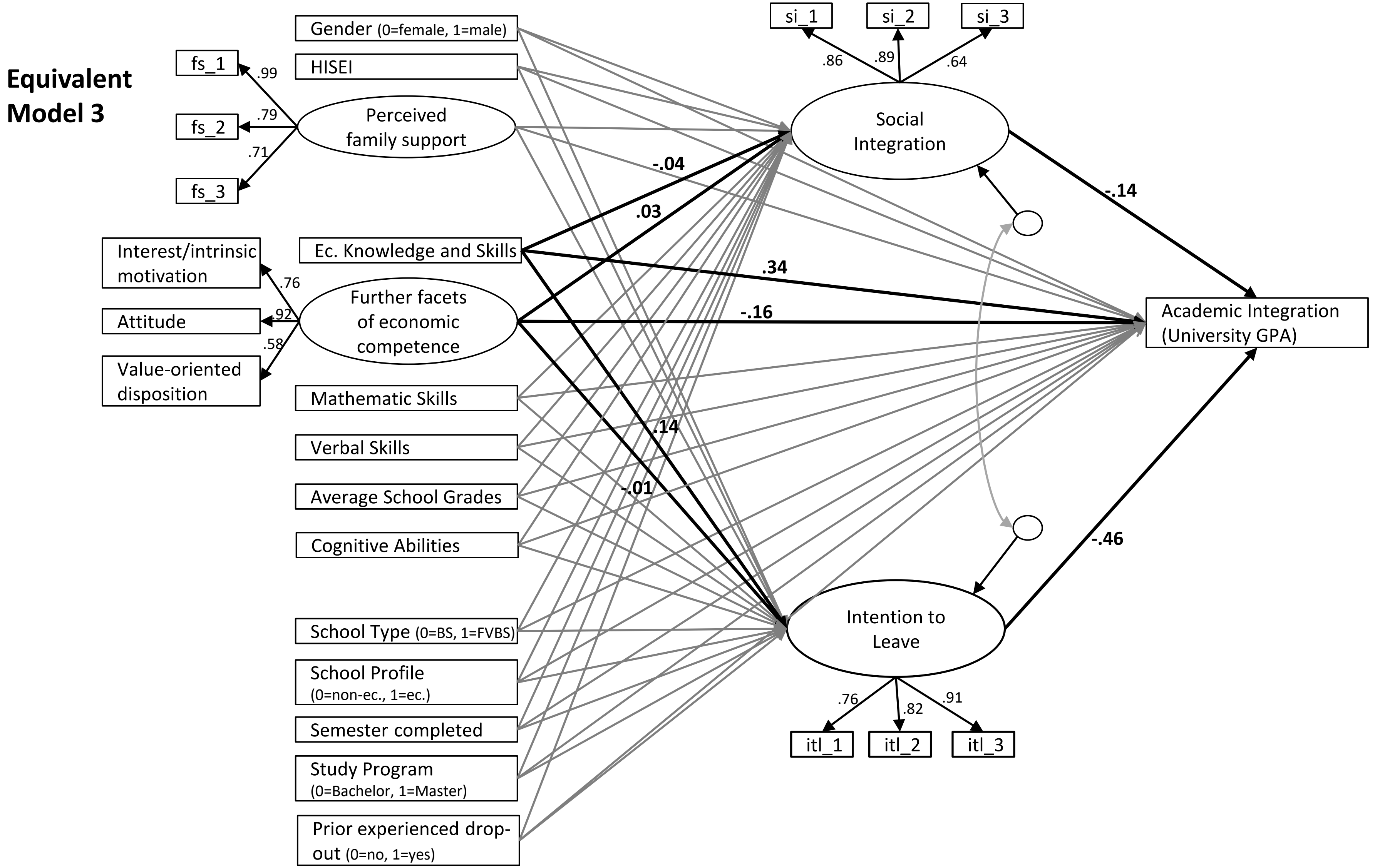
Equivalent Model 3
Gender (0=female, 1=male)
HISEI
Perceived family support
fs_1
fs_2
fs_3
.99
.79
.71
Ec. Knowledge and Skills
Interest/intrinsic motivation
Attitude
Value-oriented disposition
.76
.92
.58
Further facets of economic competence
Mathematic Skills
Verbal Skills
Average School Grades
Cognitive Abilities
School Type (0=BS, 1=FVBS)
School Profile (0=non-ec., 1=ec.)
Semester completed
Study Program (0=Bachelor, 1=Master)
Prior experienced drop-out (0=no, 1=yes)
si_1
si_2
si_3
.86
.89
.64
Social Integration
-.04
.03
.34
-.16
.14
-.01
-.14
Academic Integration (University GPA)
-.46
Intention to Leave
.76
.82
.91
itl_1
itl_2
itl_3

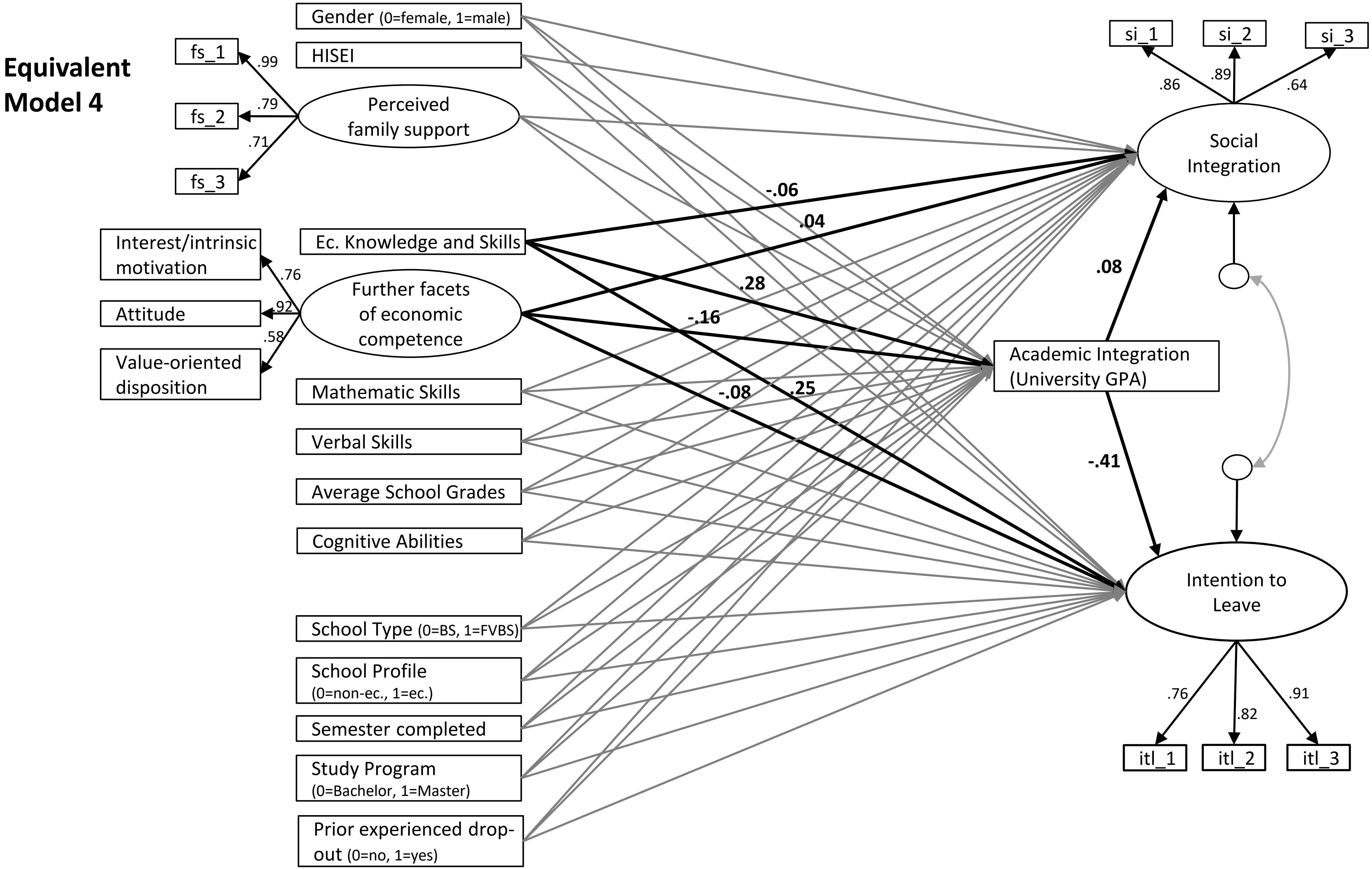

Equivalent Model 4
Gender (0=female, 1=male)
HISEI
Perceived family support
fs_1
fs_2
fs_3
.99
.79
.71
Ec. Knowledge and Skills
Interest/intrinsic motivation
Attitude
Value-oriented disposition
.76
.92
.58
Further facets of economic competence
Mathematic Skills
Verbal Skills
Average School Grades
Cognitive Abilities
School Type (0=BS, 1=FVBS)
School Profile (0=non-ec., 1=ec.)
Semester completed
Study Program (0=Bachelor, 1=Master)
Prior experienced drop-out (0=no, 1=yes)
-.06
.04
.28
-.16
-.08
.25
si_1
si_2
si_3
.86
.89
.64
Social Integration
.08
Academic Integration (University GPA)
-.41
Intention to Leave
.76
.82
.91
itl_1
itl_2
itl_3

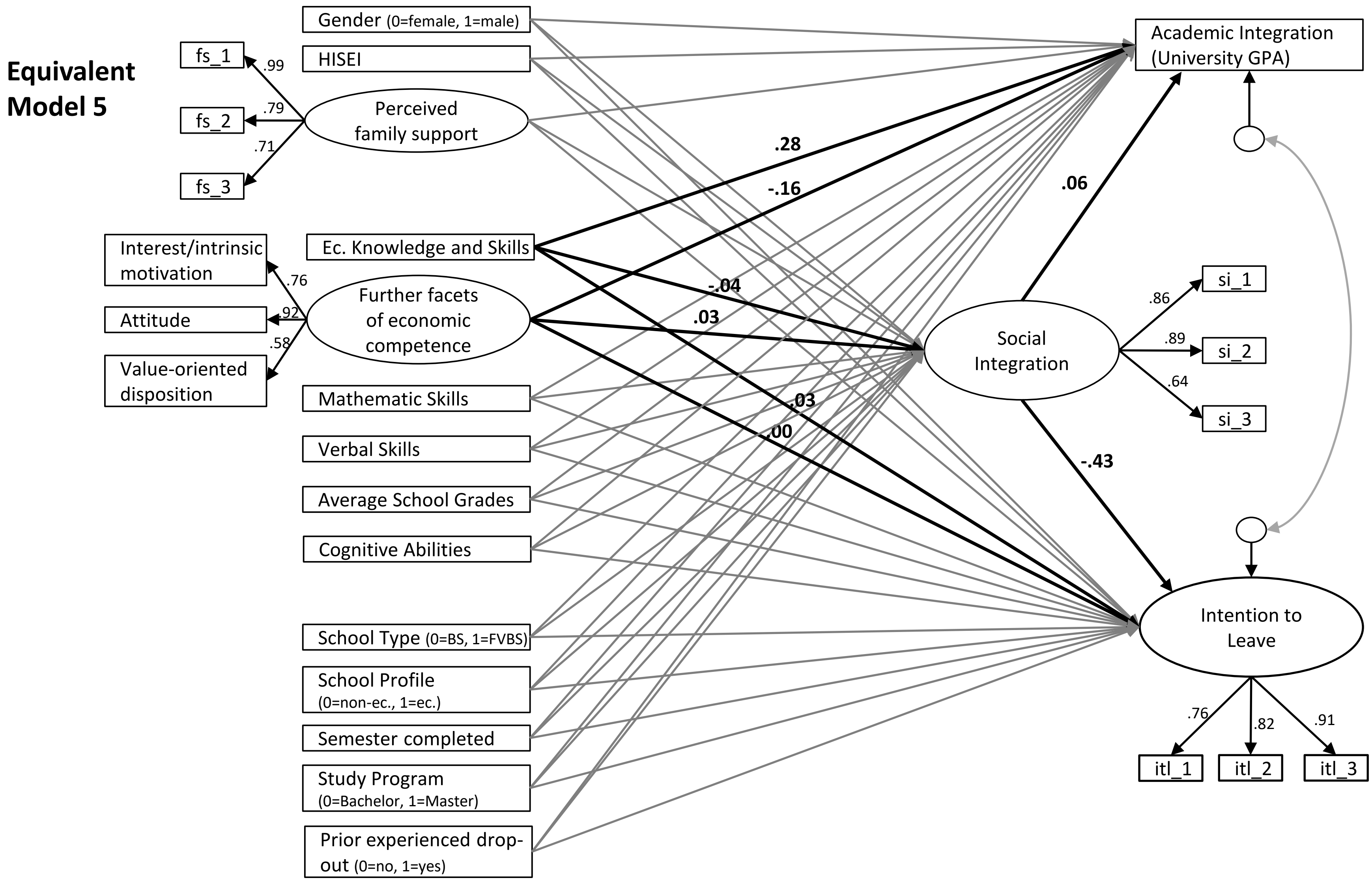

Equivalent Model 5
fs_1
fs_2
fs_3
.99
.79
.71
Perceived family support
Gender (0=female, 1=male)
HISEI
Interest/intrinsic motivation
Attitude
Value-oriented disposition
.76
.92
.58
Further facets of economic competence
Ec. Knowledge and Skills
Mathematic Skills
Verbal Skills
Average School Grades
Cognitive Abilities
School Type (0=BS, 1=FVBS)
School Profile (0=non-ec., 1=ec.)
Semester completed
Study Program (0=Bachelor, 1=Master)
Prior experienced drop-out (0=no, 1=yes)
.28
-.16
-.04
.03
.03
.00
Academic Integration (University GPA)
.06
Social Integration
.86
.89
.64
si_1
si_2
si_3
-.43
Intention to Leave
.76
.82
.91
itl_1
itl_2
itl_3
