# Supplementary material for: Predicting economics student retention in higher education: The effects of students’ economic competencies at the end of upper secondary school on their intention to leave their studies in economics
Source: PLoS One. 2020 Feb 5;15(2):e0228505. doi: 10.1371/journal.pone.0228505 (PMC7001938; doi:10.1371/journal.pone.0228505)
Supplement: S1 File — (ZIP) [file pone.0228505.s002.zip › S2 Table_Original Model.pdf]

S2 Table. Original Model

Table 1. Direct effects

	Exogenous Variables \ Endogenous Variables	Academic Integration (UGPA)	Social Integration	Intention to Leave
(Family) Background	HISEI	.07 (.08)	.05 (.08)	-.11 (.09)
	Perceived Support from Family	-.01 (.10)	**.21† (.12)**	.02 (.12)
	Gender (0=Female, 1=Male)	-.14 (.12)	-.06 (.14)	**-.18† (.10)**
Skills and Abilities	Economic Knowledge and Skills	**.28* (.14)**	-.04 (.18)	.23 (.18)
	Psychological Disposition Related to Economic Competence	-.15 (.16)	.03 (.19)	-.06 (.16)
	Mathematics Skills	-.24 (.18)	-.14 (.21)	.08 (.17)
	Verbal Skills	-.03 (.07)	.01 (.10)	.04 (.08)
	Cognitive Abilities	-.03 (.15)	.08 (.19)	**-.30* (.15)**
Prior Schooling and Experiences	Prior experienced dropout (0=retained, 1=dropped out)	**-.28** (.11)**	**-.45** (.12)**	.08 (.12)
	Study Program (0=Bachelor's, 1=Master's)	.05 (.12)	-.26 (.18)	-.13 (.14)
	Semester (13)	-.09 (.12)	-.09 (.17)	-.14 (.12)
	Average School Grades	**.30* (.13)**	-.10 (.16)	.22 (.14)
	Advanced Course (0=non-economic, 1=economic)	-.03 (.12)	.02 (.16)	-.13 (.10)
	School Type (0=BS, 1=FVBS)	**-.26† (.15)**	**-.27† (.16)**	.07 (.13)
Mediators	Academic Integration (UGPA)	--	--	**-.38** (.16)**
	Social Integration	--	--	**-.41** (.09)**
Adjusted R-Square		*.39*	*.30*	*.59*

Model fit information: χ^2=188, df=136, CFI=0.928, RMSEA=0.050, SRMR=0.050

**p<0.01, *p<0.05, †p<0.10; significant results are highlighted in bold

HISEI: Highest International Socio-Economic Index of Occupational Status (by family), BS: Baccalaureate School, FVBS: Federal Vocational Baccalaureate School, UGPA: university grade point average

Table 2. Indirect and total effects on the intention to leave

Independent Variable	Indirect Effect (UGPA)	Indirect Effect (social integration)	Total Indirect Effect	Total Effect
Economic Knowledge and Skills	**-.11† (.07)**	.02 (.08)	-.09 (.02)	.14 (.17)
Average School Grades	**-.11† (.07)**	.04 (.07)	-.07 (.11)	.15 (.14)
Cognitive Abilities	.01 (.06)	-.03 (.08)	-.02 (.11)	**-.32* (.16)**
Prior experienced dropout (0=retained, 1=dropped out)	.11 (.07)	**.18** (.07)**	**.29** (.10)**	**.38** (.11)**
School Type (0=BS, 1=FVBS)	.10 (.08)	.11 (.08)	**.21† (.12)**	**.28* (.13)**
Perceived Support from Family	<.01 (.04)	**-.09† (.05)**	-.08 (.07)	-.06 (.12)

BS: Baccalaureate School, FVBS: Federal Vocational Baccalaureate School
**p<0.01, *p<0.05, †p<0.10; significant results are highlighted in bold
