# Supplementary material for: Predicting economics student retention in higher education: The effects of students’ economic competencies at the end of upper secondary school on their intention to leave their studies in economics
Source: PLoS One. 2020 Feb 5;15(2):e0228505. doi: 10.1371/journal.pone.0228505 (PMC7001938; doi:10.1371/journal.pone.0228505)
Supplement: S1 File — (ZIP) [file pone.0228505.s002.zip › S3 Table_Equivalent Model 1.pdf]

S3 Table. Equivalent Model 1

Table 1. Direct effects

	Exogenous Variables \ Endogenous Variables	Intention to Leave	Social Integration	Academic Integration (UGPA)
**(Family) Background**	HISEI	**-.15† (.09)**	-.02 (.09)	.01 (.08)
	Perceived Support from Family	-.06 (.12)	.18 (.12)	-.03 (.10)
	Gender (0=Female, 1=Male)	-.10 (.09)	-.11 (.13)	-.18 (.12)
**Skills and Abilities**	Economic Knowledge and Skills	.14 (.18)	.03 (.17)	**.34** (.13)**
	Psychological Disposition Related to Economic Competence	-.01 (.20)	.03 (.15)	-.16 (.16)
	Mathematics Skills	.23 (.16)	-.02 (.21)	-.15 (.18)
	Verbal Skills	.04 (.08)	.04 (.10)	-.01 (.07)
	Cognitive Abilities	**-.32† (.16)**	-.07 (.18)	-.16 (.15)
**Prior Schooling and Experiences**	Prior experienced dropout (0=retained, 1=dropped out)	**.38** (.12)**	**-.27* (.14)**	-.14 (.10)
	Study Program (0=Bachelor's, 1=Master's)	-.05 (.17)	**-.28* (.14)**	.03 (.13)
	Semester (13)	-.07 (.15)	-.12 (.14)	-.12 (.12)
	Average School Grades	.15 (.19)	-.03 (.13)	**.36** (.12)**
	Advanced Course (0=non-economic, 1=economic)	-.13 (.13)	-.05 (.12)	-.08 (.10)
	School Type (0=BS, 1=FVBS)	**.28* (.14)**	-.13 (.16)	-.15 (.13)
**Mediator**	Intention to Leave		**-.48** (.18)**	**-.40* (.16)**
Adjusted R-Square		*.37*	*.44*	*.49*

Model fit information: χ^2=205.7, df=152, CFI=0.923, RMSEA=0.050, SRMR=0.049
**p<0.01, *p<0.05, †p<0.10; significant results are highlighted in bold
HISEI: Highest International Socio-Economic Index of Occupational Status (by family), BS: Baccalaureate School, FVBS: Federal Vocational Baccalaureate School, UGPA: university grade point average

Table 2. Indirect and total effects on UGPA (regarding the intention to leave)

Independent Variable	Indirect Effect	Total Effect
Economic Knowledge and Skills	-.05 (.08)	**.28* (.14)**
Average School Grades	-.06 (.08)	**.30* (.13)**
Cognitive Abilities	.13 (.09)	-.03 (.15)
Prior experienced dropout<br>(0=retained, 1=dropped out)	.11 (.07)	.38** (.11)
School Type<br>(0=BS, 1=FVBS)	-.11 (.08)	**-.26† (.15)**

BS: Baccalaureate School, FVBS: Federal Vocational Baccalaureate School
**p<0.01, *p<0.05, †p<0.10; significant results are highlighted in bold

Table 3. Indirect and total effects on social integration (regarding the intention to leave)

**Independent Variable**	**Indirect Effect**	**Total Effect**
Economic Knowledge and Skills	-.07 (.08)	-.04 (.18)
Average School Grades	-.07 (.11)	-.10 (.16)
Cognitive Abilities	.15 (.11)	.08 (.19)
Prior experienced dropout (0=retained, 1=dropped out)	**-.18* (.08)**	**-.45** (.12)**
School Type (0=BS, 1=FVBS)	-.14 (.09)	**.27† (.16)**
HISEI	**.08† (.04)**	.05 (.08)

BS: Baccalaureate School, FVBS: Federal Vocational Baccalaureate School
**p<0.01, *p<0.05, †p<0.10; significant results are highlighted in bold
