# Supplementary material for: Predicting economics student retention in higher education: The effects of students’ economic competencies at the end of upper secondary school on their intention to leave their studies in economics
Source: PLoS One. 2020 Feb 5;15(2):e0228505. doi: 10.1371/journal.pone.0228505 (PMC7001938; doi:10.1371/journal.pone.0228505)
Supplement: S1 File — (ZIP) [file pone.0228505.s002.zip › S4 Table_Equivalent Model 2.pdf]

S4 Table. Equivalent Model 2

Table 1. Direct effects

	Exogenous Variables \ Endogenous Variables	Academic Integration (UGPA)	Intention to Leave	Social Integration
**(Family) Background**	HISEI	.07 (.08)	**-.15† (.09)**	-.02 (.09)
	Perceived Support from Family	-.01 (.10)	.06 (.12)	.02 (.12)
	Gender (0=Female, 1=Male)	-.14 (.12)	**-.10† (.09)**	-.14 (.14)
**Skills and Abilities**	Economic Knowledge and Skills	**.28* (.14)**	.14 (.17)	.08 (.18)
	Psychological Disposition Related to Economic Competence	-.15 (.16)	.01 (.20)	<.01 (.20)
	Mathematics Skills	-.24 (.18)	.23 (.16)	-.05 (.21)
	Verbal Skills	-.03 (.07)	.04 (.08)	.03 (.10)
	Cognitive Abilities	-.03 (.15)	**-.32† (.16)**	-.10 (.18)
**Prior Schooling and Experiences**	Prior experienced dropout (0=retained, 1=dropped out)	**-.28* (.11)**	**.38** (.12)**	**-.29* (.14)**
	Study Program (0=Bachelor's, 1=Master's)	.05 (.12)	.05 (.17)	**-.28* (.14)**
	Semester (13)	-.09 (.12)	-.06 (.15)	-.14 (.15)
	Average School Grades	**.30* (.13)**	.15 (.19)	.02 (.16)
	Advanced Course (0=non-economic, 1=economic)	-.03 (.12)	-.13 (.13)	-.06 (.12)
	School Type (0=BS, 1=FVBS)	**-.26† (.15)**	**.28† (.14)**	-.15 (.17)
**Mediators**	Academic Integration (UGPA)	--	--	-.15 (.15)
	Intention to Leave	--	--	**-.54** (.19)**
Adjusted R-Square		*.39*	*.37*	*.46*

Model fit information: χ^2=205.7, df=152, CFI=0.923, RMSEA=0.050, SRMR=0.049
**p<0.01, *p<0.05, †p<0.10; significant results are highlighted in bold
HISEI: Highest International Socio-Economic Index of Occupational Status (by family), BS: Baccalaureate School, FVBS: Federal Vocational Baccalaureate School, UGPA: university grade point average

Table 2. Indirect and total effects on social integration

**Independent Variable**	**Indirect Effect (UGPA)**	**Indirect Effect (intention to leave)**	**Total Indirect Effect**	**Total Effect**
Economic Knowledge and Skills	-.04 (.05)	-.07 (.09)	-.12 (.09)	-.04 (.18)
Average School Grades	-.04 (.05)	-.08 (.07)	-.13 (.12)	-.10 (.16)
Cognitive Abilities	.01 (.03)	.17 (.12)	.18 (.12)	.08 (.19)
Prior experienced dropout (0=retained, 1=dropped out)	.04 (.05)	**-.20* (.10)**	**-.16† (.09)**	**-.45** (.12)**
School Type (0=BS, 1=FVBS)	.04 (.10)	-.15 (.10)	-.11 (.10)	**-.27† (.16)**
HISEI	-.01 (.02)	**.08† (.05)**	.07 (.05)	.05 (.08)
Gender (0=Female, 1=Male)	.02 (.03)	.06 (.05)	.08 (.06)	-.06 (.14)

BS: Baccalaureate School, FVBS: Federal Vocational Baccalaureate School
**p<0.01, *p<0.05, †p<0.10; significant results are highlighted in bold
