# Supplementary material for: Predicting economics student retention in higher education: The effects of students’ economic competencies at the end of upper secondary school on their intention to leave their studies in economics
Source: PLoS One. 2020 Feb 5;15(2):e0228505. doi: 10.1371/journal.pone.0228505 (PMC7001938; doi:10.1371/journal.pone.0228505)
Supplement: S1 File — (ZIP) [file pone.0228505.s002.zip › S5 Table_Equivalent Model 3.pdf]

S5 Table. Equivalent Model 3

Table 1. Direct effects

	Exogenous Variables \ Endogenous Variables	Intention to Leave	Social Integration	Academic Integration (UGPA)
**(Family) Background**	HISEI	**-.15† (.09)**	.05 (.08)	.01 (.08)
	Perceived Support from Family	.06 (.12)	**.21† (.12)**	-.01 (.10)
	Gender (0=Female, 1=Male)	**-.10† (.09)**	-.06 (.14)	**-.18† (.10)**
**Skills and Abilities**	Economic Knowledge and Skills	.14 (.17)	-.04 (.18)	**.34* (.13)**
	Psychological Disposition Related to Economic Competence	.01 (.20)	.03 (.19)	-.16 (.16)
	Mathematics Skills	.23 (.16)	-.14 (.21)	-.15 (.18)
	Verbal Skills	.04 (.08)	.01 (.10)	-.01 (.07)
	Cognitive Abilities	**-.32† (.16)**	.08 (.19)	-.17 (.16)
**Prior Schooling and Experiences**	Prior experienced dropout (0=retained, 1=dropped out)	**.38** (.12)**	**-.45** (.12)**	.08 (.12)
	Study Program (0=Bachelor's, 1=Master's)	.05 (.17)	-.26 (.18)	-.13 (.14)
	Semester (13)	-.06 (.15)	-.09 (.17)	-.13 (.13)
	Average School Grades	.15 (.19)	-.10 (.16)	**.35** (.12)**
	Advanced Course (0=non-economic, 1=economic)	-.13 (.13)	.02 (.16)	-.13 (.10)
	School Type (0=BS, 1=FVBS)	**.28† (.14)**	**-.27† (.16)**	.07 (.13)
**Mediators**	Intention to Leave	--	--	**-.46** (.17)**
	Social Integration	--	--	-.14 (.14)
Adjusted R-Square		*.37*	*.30*	*.50*

Model fit information: χ^2=205.7, df=152, CFI=0.923, RMSEA=0.050, SRMR=0.049
**p<0.01, *p<0.05, †p<0.10; significant results are highlighted in bold
HISEI: Highest International Socio-Economic Index of Occupational Status (by family), BS: Baccalaureate School, FVBS: Federal Vocational Baccalaureate School, UGPA: university grade point average

Table 2. Indirect and total effects on academic integration (UGPA)

Independent Variable	Indirect Effect (intention to leave)	Indirect Effect (social integration)	Total Indirect Effect	Total Effect
Economic Knowledge and Skills	-.06 (.08)	.01 (.03)	-.05 (.08)	**.28* (.17)**
Average School Grades	-.07 (.09)	.01 (.03)	-.06 (.08)	**.30* (.13)**
Cognitive Abilities	.15 (.10)	-.01 (.03)	.14 (.09)	-.03 (.15)
Prior experienced dropout (0=retained, 1=dropped out)	**-.17† (.09)**	.06 (.06)	-.11 (.10)	**-.28* (.11)**
School Type (0=BS, 1=FVBS)	-.13 (.10)	.04 (.05)	.09 (.09)	**.26† (.09)**
Perceived Support from Family	.03 (.05)	-.03 (.03)	.00 (.06)	-.01 (.10)
HISEI	.07 (.04)	-.01 (.01)	.06 (.04)	.07 (.08)
Gender (0=Female, 1=Male)	.05 (.04)	.01 (.02)	.06 (.04)	-.14 (.12)

BS: Baccalaureate School, FVBS: Federal Vocational Baccalaureate School
**p<0.01, *p<0.05, †p<0.10; significant results are highlighted in bold
