# Supplementary material for: Predicting economics student retention in higher education: The effects of students’ economic competencies at the end of upper secondary school on their intention to leave their studies in economics
Source: PLoS One. 2020 Feb 5;15(2):e0228505. doi: 10.1371/journal.pone.0228505 (PMC7001938; doi:10.1371/journal.pone.0228505)
Supplement: S1 File — (ZIP) [file pone.0228505.s002.zip › S6 Table_Equivalent Model 4.pdf]

S6 Table. Equivalent Model 4

Table 1. Direct effects

	Exogenous Variables \ Endogenous Variables	Academic Integration (UGPA)	Social Integration	Intention to Leave
(Family) Background	HISEI	.07 (.08)	.05 (.08)	-.13 (.09)
	Perceived Support from Family	-.01 (.10)	**.21† (.12)**	-.06 (.12)
	Gender (0=Female, 1=Male)	-.14 (.12)	-.06 (.15)	-.16 (.10)
Skills and Abilities	Economic Knowledge and Skills	**.28* (.14)**	-.06 (.18)	.25 (.18)
	Psychological Disposition Related to Economic Competence	-.15 (.16)	.04 (.20)	-.08 (.19)
	Mathematics Skills	-.24 (.18)	-.12 (.21)	.13 (.17)
	Verbal Skills	-.03 (.07)	.02 (.11)	.03 (.08)
	Cognitive Abilities	-.03 (.15)	.08 (.19)	**-.33* (.16)**
Prior Schooling and Experiences	Prior experienced dropout (0=retained, 1=dropped out)	**-.28** (.11)**	**-.43** (.13)**	**.26* (.11)**
	Study Program (0=Bachelor's, 1=Master's)	.05 (.12)	-.26 (.18)	-.02 (.17)
	Semester (13)	-.09 (.12)	-.08 (.17)	-.10 (.14)
	Average School Grades	**.30* (.13)**	-.13 (.18)	**.28† (.16)**
	Advanced Course (0=non-economic, 1=economic)	-.03 (.12)	.02 (.15)	-.14 (.12)
	School Type (0=BS, 1=FVBS)	**-.26† (.15)**	-.25 (.17)	.17 (.12)
Mediator	Academic Integration (UGPA)	--	.08 (18)	**-.41* (.18)**
Adjusted R-Square		*.39*	*.30*	*.47*

Model fit information: χ^2=205.7, df=152, CFI=0.923, RMSEA=0.050, SRMR=0.049
**p<0.01, *p<0.05, †p<0.10; significant results are highlighted in bold
HISEI: Highest International Socio-Economic Index of Occupational Status (by family), BS: Baccalaureate School, FVBS: Federal Vocational Baccalaureate School, UGPA: university grade point average

Table 2. Indirect and total effects on the intention to leave (regarding academic integration)

**Independent Variable**	**Indirect Effect**	**Total Effect**
Economic Knowledge and Skills	**-.12† (.07)**	.14 (.17)
Average School Grades	**-.12† (.08)**	.15 (.19)
Cognitive Abilities	.02 (.06)	**-.32† (.16)**
Prior experienced dropout (0=retained, 1=dropped out)	.12 (.08)	**.38** (.12)**
School Type (0=BS, 1=FVBS)	.11 (.09)	**.28* (.13)**
Perceived Support from Family	.00 (.04)	-.06 (.12)

BS: Baccalaureate School, FVBS: Federal Vocational Baccalaureate School
**p<0.01, *p<0.05, †p<0.10; significant results are highlighted in bold

Table 3. Indirect and total effects on social integration (regarding academic integration)

Independent Variable	Indirect Effect	Total Effect
Economic Knowledge and Skills	.02 (.05)	-.04 (.18)
Average School Grades	.04 (.07)	.15 (.14)
Cognitive Abilities	-.01 (.02)	.07 (.19)
Prior experienced dropout (0=retained, 1=dropped out)	-.02 (.06)	**.45** (.12)**
School Type (0=BS, 1=FVBS)	-.02 (.05)	**.27† (.16)**
Perceived Support from Family	.00 (.01)	**.21† (.12)**

BS: Baccalaureate School, FVBS: Federal Vocational Baccalaureate School
**p<0.01, *p<0.05, †p<0.10; significant results are highlighted in bold
