# Supplementary material for: Predicting economics student retention in higher education: The effects of students’ economic competencies at the end of upper secondary school on their intention to leave their studies in economics
Source: PLoS One. 2020 Feb 5;15(2):e0228505. doi: 10.1371/journal.pone.0228505 (PMC7001938; doi:10.1371/journal.pone.0228505)
Supplement: S1 File — (ZIP) [file pone.0228505.s002.zip › S7 Table_Equivalent Model 5.pdf]

S7 Table. Equivalent Model 5

Table 1. Direct effects

	Exogenous Variables \ Endogenous Variables	**Social Integration**	**Academic Integration (UGPA)**	**Intention to Leave**
**(Family) Background**	HISEI	.05 (.08)	.07 (.08)	-.13 (.09)
	Perceived Support from Family	**.21† (.12)**	-.02 (.10)	.03 (.12)
	Gender (0=Female, 1=Male)	-.06 (.14)	-.14 (.12)	**-.18† (.10)**
**Skills and Abilities**	Economic Knowledge and Skills	-.04 (.18)	**.29* (.14)**	.12 (.17)
	Psychological Disposition Related to Economic Competence	.03 (.19)	-.16 (.17)	.00 (.16)
	Mathematics Skills	-.14 (.21)	-.23 (.18)	.17 (.16)
	Verbal Skills	.01 (.10)	-.03 (.07)	.05 (.09)
	Cognitive Abilities	.08 (.19)	-.04 (.15)	**-.29* (.15)**
**Prior Schooling and Experiences**	Prior experienced dropout (0=retained, 1=dropped out)	**-.45** (.12)**	**-.26* (.12)**	.18 (.13)
	Study Program (0=Bachelor's, 1=Master's)	-.26 (.18)	.07 (.13)	-.16 (.14)
	Semester (13)	-.09 (.17)	-.08 (.12)	-.10 (.13)
	Average School Grades	-.10 (.16)	**.30* (.13)**	.11 (.17)
	Advanced Course (0=non-economic, 1=economic)	.02 (.16)	-.04 (.11)	-.12 (.10)
	School Type (0=BS, 1=FVBS)	**-.27† (.16)**	**-.24† (.15)**	.16 (.13)
**Mediators**	Social Integration	--	.06 (.16)	**-.43** (.11)**
Adjusted R-Square		*.30*	*.40*	*.50*

Model fit information: χ^2=205.7, df=152, CFI=0.923, RMSEA=0.050, SRMR=0.049
**p<0.01, *p<0.05, †p<0.10; significant results are highlighted in bold
HISEI: Highest International Socio-Economic Index of Occupational Status (by family), BS: Baccalaureate School, FVBS: Federal Vocational Baccalaureate School, UGPA: university grade point average

Table 2. Indirect and total effects on UGPA (regarding social integration)

**Independent Variable**	**Indirect Effect**	**Total Effect**
Economic Knowledge and Skills	.00 (.02)	**.28* (.14)**
Average School Grades	-.01 (.02)	**.30* (.13)**
Cognitive Abilities	.00 (.03)	-.03 (.15)
Prior experienced dropout (0=retained, 1=dropped out)	-.03 (.07)	**-.28* (.11)**
School Type (0=BS, 1=FVBS)	-.02 (.04)	**-.26† (.15)**
Perceived Support from Family	.01 (.04)	-.01 (.10)

BS: Baccalaureate School, FVBS: Federal Vocational Baccalaureate School
**p<0.01, *p<0.05, †p<0.10; significant results are highlighted in bold

Table 3. Indirect and total effects on the intention to leave (regarding social integration)

**Independent Variable**	**Indirect Effect (Social Integration)**	**Total Effect**
Economic Knowledge and Skills	.02 (.08)	.14 (.17)
Average School Grades	.04 (.07)	.15 (.14)
Cognitive Abilities	-.03 (.08)	**-.32* (.16)**
Prior experienced dropout (0=retained, 1=dropped out)	**.18** (.07)**	**.38** (.11)**
School Type (0=BS, 1=FVBS)	.11 (.08)	**.28* (.13)**
Perceived Support from Family	**-.09† (.05)**	-.06 (.12)

BS: Baccalaureate School, FVBS: Federal Vocational Baccalaureate School
**p<0.01, *p<0.05, †p<0.10; significant results are highlighted in bold
